# Biocompatible Solvents and Ionic Liquid-Based Surfactants as Sustainable Components to Formulate Environmentally Friendly Organized Systems

**DOI:** 10.3390/polym13091378

**Published:** 2021-04-23

**Authors:** Nahir Dib, Cristian M. O. Lépori, N. Mariano Correa, Juana J. Silber, R. Dario Falcone, Luis García-Río

**Affiliations:** 1 Departamento de Química, Universidad Nacional de Río Cuarto (UNRC), Agencia Postal No. 3, X5804BYA Río Cuarto, Argentina; ndib@exa.unrc.edu.ar (N.D.); mcorrea@exa.unrc.edu.ar (N.M.C.); jsilber@exa.unrc.edu.ar (J.J.S.); 2 Instituto para el Desarrollo Agroindustrial y de la Salud (IDAS), CONICET–UNRC, Agencia Postal No. 3, X5804BYA Río Cuarto, Argentina; 3 Instituto de Física Enrique Gaviola (IFEG), Consejo Nacional de Investigaciones Científicas y Técnicas (CONICET)–Universidad Nacional de Córdoba (UNC), Medina Allende s/n, Ciudad Universitaria, X5016LAE Córdoba, Argentina; clepori@exa.unrc.edu.ar; 4 Centro Singular de Investigación en Química Biolóxica e Materiais Moleculares (CIQUS), Departamento de Química Física, Universidade de Santiago de Compostela, 15782 Santiago, Spain

**Keywords:** reverse micelles, ionic liquids, surfactant, biocompatible, IPM

## Abstract

In this review, we deal with the formation and application of biocompatible water-in-oil microemulsions commonly known as reverse micelles (RMs). These RMs are extremely important to facilitate the dissolution of hydrophilic and hydrophobic compounds for biocompatibility in applications in drug delivery, food science, and nanomedicine. The combination of two wisely chosen types of compounds such as biocompatible non-polar solvents and ionic liquids (ILs) with amphiphilic character (surface-active ionic liquids, SAILs) can be used to generate organized systems that perfectly align with the Green Chemistry concepts. Thus, we describe the current state of SAILs (protic and aprotic) to prepare RMs using non-polar but safe solvents such as esters derived from fatty acids, among others. Moreover, the use of the biocompatible solvents as the external phase in RMs and microemulsions/nanoemulsions with the other commonly used biocompatible surfactants is detailed showing the diversity of preparations and important applications. As shown by multiple examples, the properties of the RMs can be modified by changes in the type of surfactant and/or external solvents but a key fact to note is that all these modifications generate novel systems with dissimilar properties. These interesting properties cannot be anticipated or extrapolated, and deep analysis is always required. Finally, the works presented provide valuable information about the use of biocompatible RMs, making them a green and promising alternative toward efficient and sustainable chemistry.

## 1. Introduction

**Scheme 1:**
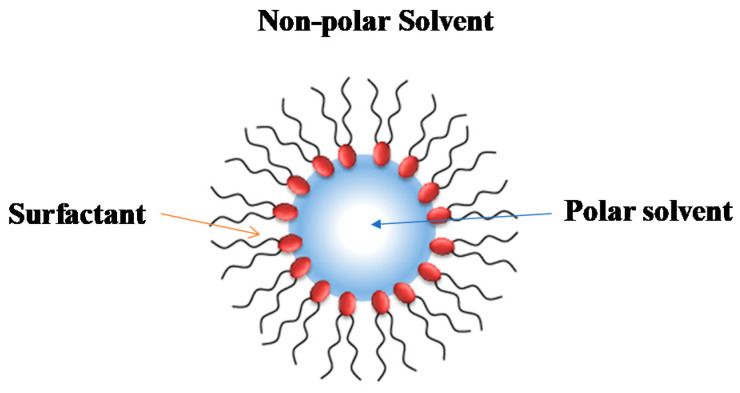
Schematic representation of a typical reverse micelles (RMs) structure.

When certain surfactants, molecules that have in their moiety a polar and a non-polar part, are dissolved in a non-polar organic solvent, they arrange themselves, forming aggregates known as reverse micelles (RMs) [1,2]. Thus, the surfactants′ non-polar part (typically, a long hydrocarbon chain) points toward the organic solvent, while the polar part (which can be salts with its counterions or even polar noncharged groups) point far from the non-polar environment, interacting with each other and forming the polar core of the RMs (See Scheme 1) [3,4].

RMs are organized systems coexisting in water-in-oil (W/O) microemulsions. In this sense, both definitions (RMs and microemulsions) are normally used as synonyms but are not equal. The concept of the microemulsion applies to a liquid solution composed of water, oil, and an amphiphile (surfactant and cosurfactant) forming a single, optically isotropic and thermodynamically stable phase [5]. Thus, a microemulsion shows a certain degree of intermolecular arrangement but without a well-defined stoichiometric composition. On the other hand, RM solutions are systems with a strong molecular organization [1].

The term nanoemulsion is also used to describe an isotropic, transparent mixture of two immiscible liquids, i.e., oil and water. In contrast to microemulsions, nanoemulsions are thermodynamically unstable systems; however, the kinetics of destabilization are very slow and these can be considered kinetically stable systems [6]. Depending on the oil/water ratio, there are oil-in-water (O/W), water-in-oil (W/O), and bi-continuous nanoemulsions (also known as multiple emulsions) which are all stabilized by the use of a mixture of a surfactant and/or cosurfactant. The O/W and W/O dispersions form droplets of nanometric sizes smaller than 300 nm, typically in the range of 20 to 200 nm [6,7]. There are two types of preparation methods: (i) One uses high-energy emulsification systems to increase the surface area by size reduction and includes ultra-sonification, using micro fluidizers and high-pressure homogenizers. (ii) The other is low-energy emulsification methods, which depend on the physicochemical properties of the compounds and include phase inversion temperature, solvent displacement, and phase inversion methods. In their uses and applications, nanoemulsions and microemulsions have similarities and differences, and these have been exhaustively reviewed [6,8,9].

The polar core of the RMs can be dissolved in highly polar molecules that are non-soluble in non-polar organic solvents including water. These nanoscopic water or solvent pools can be used for a range of applications from nanoparticle and polymer synthesis, to the enhancement of chemical reaction rates and even to models for water in biological confinement since the bulk water properties are deeply affected because of the confinement to a nanoscopic scale. Thus, the microviscosity, micropolarity, hydrogen bond abilities, electron donor, or acceptor properties are deeply modified [1,2].

Several different surfactants are used to form different kinds of aggregates when dissolved in water, but only a few of them can form RMs. The majority of the investigations have focused on systems that utilize Na-aerosol OT (sodium diethylhexyl sulfosuccinate, sodium docusate, Na-AOT) as the amphiphile. Na-AOT can form RMs in a wide range of non-polar solvents such as alkanes, aromatic solvents, haloalkanes, supercritical alkane solutions, and ionic liquids without the use of any co-surfactant. The sizes of the Na-AOT RMs depend on the water-to-surfactant ratio, W_0_ = [H_2_O]/[surfactant], the non-polar solvent, and the temperature [1].

Among the wide range of applications of RMs, one very important is the use of them as nano templates for nano synthesis, such as nanoparticles or nanopolymers. The basis for those syntheses is founded on the droplet–droplet interactions. The rate at which the reactants pass from one RM droplet to the other is the key to nano synthesis. If the droplet–droplet interaction is favored, the nanoparticles will have a larger size than the case where the droplet–droplet interaction is inhibited. Further, as the synthesis occurs in the RM water pool, the confinement makes it impossible for the particles to grow further than the nanometer scale [10,11].

On the other hand, the traditional RM systems have a problem with the environment, thinking about applying them to an industrial scale. The conventional non-polar solvents used are mainly toxic and/or non-environmentally friendly. One strategy to overcome this is the use of the proper ionic liquids (ILs) [12,13]. ILs are compounds that are composed entirely of ions and, because of the large size of the anion or cation or both, the melting point is low in comparison with traditional salts. Accordingly, some of them are liquid at room temperature (room temperature ionic liquids, RTILs) while others have a melting point below 100^°^ C. Several unique properties make them an excellent choice to be used as solvents. For example, they have negligible vapor pressure at ambient temperature, they have excellent chemical and thermal stability, they are easy to recycle, as well as other properties such as polarity, solvent miscibility, hydrophobicity which are highly tunable through the appropriate selection of the cation and the anion [14,15,16,17,18,19,20].

Another interesting feature of the ILs is that their amphiphilic characteristic can be designed, thus producing ionic liquid-like surfactants (IL-like surfactants) or simply surface-active ionic liquids (SAILs). These compounds can be used as environmentally friendly surfactants as we discuss in the next section [21].

## 2. Surface-Active Ionic Liquids (SAILs)

**Scheme 2:**
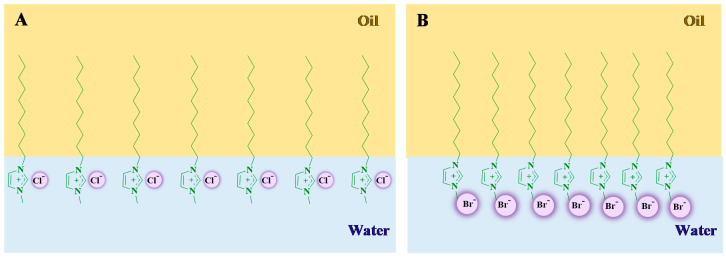
Cartoon representation of the effect of polarizability between imidazolium-type surface-active ionic liquids (SAILs) with Cl^−^ (**A**) or Br^−^ (**B**) anions resulting in a different monomeric arrangements at the oil–water interface. The presence of the Br^−^ counterion induces less repulsion between SAIL monomers due to higher polarizability of Br^−^ leading to the formation of a more stable layer at the oil–water interface. Adapted from [54]. Copyright 2020, American Chemical Society.

SAILs have certain advantages over the common surfactants in terms of the formation of a stable interface due to the favorable interactions between the cation and anion. Amphiphilic ILs are constituted by a charged hydrophilic headgroup and one or more hydrophobic tails. There is an evident structural similarity between frequently used traditional surfactants (salts) and long-chain IL (liquid salts), and hence, ILs are also able to self-aggregate as micelles in an aqueous solution, similar to common ionic amphiphiles. SAILs have inherited unique properties from ILs: low vapor pressure, high thermal stability, high ionic conductivity, and high solvating capacity for both polar and non-polar compounds and wide electrochemical windows. SAILs are capable of self-aggregation in solution which implies that, similar to surfactants, the cation or anion of some ILs might have a charged hydrophilic headgroup and a hydrophobic tail (alkyl chain) [22,23]. It has been observed that SAILs exhibit low critical micelle concentration (CMC) values when compared to conventional surfactants with a similar structure. The chemical structure of the most common SAILs as well as their CMC in water are depicted in Scheme 2. SAILs can also be designed for task-specific functions with a simple change in the structure of their anions/cations. The ability of SAILs to create organized systems such as direct and RMs or vesicles formation has opened perspectives for application in diverse fields of biotechnology, analytical chemistry, catalysis, fabrication of nanomaterials, etc.

### 2.1. SAILs in Water

Long-chain ILs composed of the 1-alkyl-3-methylimidazolium cation, [C_n_mim]^+^, are among this novel type of amphiphiles that have been studied in recent years [24,25,26]. For [C_n_mim][Cl] ILs, there is a good correlation between the Gibbs energy of micellization in water and *n*, with the type of dependence being similar to that shown by alkyltrimethylammonium and alkylammonium chloride families. Isothermal titration microcalorimetry has been used to obtain the thermodynamic parameters of micellization for [C_12_mim][Br], [C_14_mim][Br], and [C_16_mim][Br], asserting that the process of micellization is entropy-driven [27]. SAILs with imidazolium core have several advantages compared to conventionally employed surfactants; the immobilization of the (co-)surfactant at the fluid/fluid interface is promoted via its attraction force towards the imidazolium ring; the broad imidazolium head is strongly attractive through π-π interactions and has a solubilizing capacity for a broader range of solutes compared to quaternary ammonium-based cationic surfactants [28,29]. These advantages have been explored for micellar catalysis in water, demonstrating that SAILs can considerably affect the outcome of reactions [30,31,32]. Moreover, the capacity for ionic liquid structural tuning offers virtually an unlimited range of optimization options of the hydrophilic–lipophilic equilibrium-based partitioning behavior, and hence an ideal pre-requisite for the formation of ionic liquid–oil–water RMs.

A different class of IL-based surfactants is that containing double chains or a dimeric structure similar to gemini surfactants. The length of the covalent spacers between headgroups and the length of the chains can significantly affect the properties of gemini-type ILs. For instance, physicochemical properties of 1,4-bi(3-tetradecylimidazolium-1-yl) butane bromide (see Table 1) show that this novel gemini IL possesses higher thermal stability, lower CMC, and a larger d-spacing of crystal structure than the homologous ionic liquids with a single long chain [33]. Using surface tensiometry, the self-aggregation of six dicationic imidazolium-based ILs with structures resembling gemini and bola-forming surfactants has been reported [34]. Moreover, the aggregation behavior of imidazolium-based gemini surfactants has been explored and compared with the equivalent surfactants possessing ammonium head groups [35]. In the case of the IL, upon increasing the spacer chain length *s*, the CMC increases. This trend can be explained in terms of conformational changes and progressive looping of the spacer into the micellar interior upon increasing *s*. In contrast, for conventional bis-quatammonium-based cationic gemini surfactants, the CMC has a non-monotonic dependence with *s*, reaching a maximum at *s* = 6 [36].

It is well known that at surfactant concentrations lower than the CMC, surfactants form a monomeric layer at the interface. When the minimum free energy state is attained, the monomers within the aqueous solution tend to associate into direct micelles. Thus, it can be said that the basic driving force for micelle formation is the need to minimize the Gibbs free energy. This can be achieved by minimizing the degree of mixing between the hydrophobic tails of the surfactant with water and keeping the opposite condition true for its hydrophilic head [43]. Many researchers have observed that SAILs exhibit lower CMC values when compared to conventional surfactants with similar structures (see Scheme 2). It has been found that, as in the case of conventional surfactants, increasing the chain length of the hydrophobic tail strongly decreases the CMC values of SAILs [44,45,46]. The effect of the anions attached to the cationic headgroup on the CMC has been investigated. For [C_12_mim][X], where X = AcO^−^; Cl^−^; Br^−^; and I^-^ at 25 °C, it was found that the CMC values increased in the order [C_12_mim][AcO] < [C_12_mim][I] < [C_12_mim][Br] < [C_12_mim][Cl] [47]. The influence of anions on the CMC of SAILs has been attributed to hydrated radius/polarizability, hydrophobicity, and bulkiness of the anions. As the polarizability (ease with which the electron cloud of an anion can be distorted by a cation) increases, the binding of an anion to the cationic head group increases. As a result, there is less repulsion between the headgroups of the SAILs at the air–water interface that leads to a decrease in the formation of the monomer layer at the interface and subsequent lowering of CMC values [48].

The nature of the headgroup also has an important effect on the CMC value in such a way that the hydrophobicity or van der Waals volume of the cationic headgroup of the SAILs is attributed to the change in their respective CMC values [49]. Different studies have shown that SAILs having a multicationic headgroup display improved surface activity as compared to monocationic analogs [34,50]. Besides, the formation of a catanionic SAIL (surfactant composed of anionic and cationic amphiphilic components) affects the aggregation of its monomers with a high tendency for micellization at low concentrations [51].

### 2.2. SAILs in Non-Polar Solvents

As mentioned before, the formation of stable RMs between two inherently immiscible liquids such as oil and water requires the presence of surfactants to reduce the interfacial tension between the two phases. It is important to analyze the SAIL influence (alkyl chain length, type of headgroup, and type of counterion) on the interfacial tension between oil and water in comparison with conventional surfactants. The influence of the length of the hydrophobic tail of SAILs on the interfacial tension between oil and the aqueous ionic liquid solution has been widely studied.

#### 2.2.1. Imidazolium and Pyridinium-Based SAILs

Protic SAILs having longer hydrophobic tails and/or a high number of hydrophobic tails attached to the headgroup have an increased affinity toward oil; i.e., they are more lipophilic and have a more suitable HLB (hydrophilic–lipophilic balance) value [52]. Thus, more stable would be the SAIL monomers adsorbed at the oil–water interface, leading to a further reduction in the interfacial tension. Nandwani et al. studied the influence of the alkyl chain length of imidazolium-based aprotic SAILs on their interfacial tension reduction ability, showing that as the alkyl chain length increased from *n* = 12 to *n* = 16, the interfacial tension reduction capacity of the respective SAIL increased [44]. The reason behind this trend can be attributed to the increased hydrophobicity of SAILs resulting from an increase in the hydrophobic tail length. This longer hydrophobic tail could have a greater van der Waals force of attraction with oil, which can lead to the reduction of the interfacial tension between oil and water.

Aprotic SAILs derived from imidazolium and pyridinium have been used to study the interfacial tension between crude oil and water, showing that the same pyridinium-based SAILs are less effective in reducing the interfacial tension as compared to imidazolium-based SAILs (having the same alkyl chain length) [53]. Very recently, studies indicated [54] that cationic SAILs are generally more efficient in reducing the interfacial tension between oil and water than the conventional cationic surfactants having the same hydrophobic tail length. Among heterocyclic SAILs, those with multicationic, zwitterionic headgroups, or an aromatic head group are more efficient. This might be attributed to the enhanced hydrophobicity of such SAILs. Catanionic SAILs which have a long alkyl chain attached to the anionic moiety display significant interfacial tension-reducing capability as compared to conventional anionic surfactants. This is because such catanionic SAILs exhibit pronounced interfacial activity, in that they are more efficiently absorbed at the oil–water interface.

Studies on the effect of the type of anion attached in SAILs having the same headgroup and alkyl chain length on the interfacial tension reduction efficiency concluded that anions with high molecular weight, higher hydrated radius/polarizability, and high bulkiness/viscosity interact better with the crude oil moiety and thereby show a higher efficiency in reducing the interfacial tension. Anions with high polarizability or high hydrated radius generate a strong bond with the cation due to which the cation penetrates the electron cloud of the anion. As a result, they minimize repulsion between monomers at the oil–water interface and lead to further interfacial tension reduction. Scheme 2 gives a cartoon representation of polarizability between imidazolium-type SAILs and Br^−^ or Cl^−^ anions, resulting in a stable or less stable monomeric arrangement at the oil–water interface.

Although the employment of ILs as a component in RMs is still under investigation [55], some typical examples of SAIL-based RMs are summarized in Table 2. These RMs have been formulated based on alkylimidazolium surfactants.

#### 2.2.2. AOT-Based SAILs

SAILs composed of the anionic 1,4-bis(2-ethylhexyl)sulfosuccinate surfactant have been prepared in the past. Two novel catanionic SAILs, benzyl-n-hexadecyldimethylammonium 1,4-bis(2-ethylhexyl)sulfosuccinate (BHD-AOT, Table 1) and cetyltrimethylammonium 1,4-bis(2-ethylhexyl)sulfosuccinate (C_16_TA-AOT,Table 1), were formulated [39,40,64,65]. Both have shown interesting properties as surfactants to create organized systems. We have demonstrated that these new surfactants form spherical RMs in different non-polar solvents, and the magnitude of the water–catanionic surfactant interaction at the interface is different in both systems. In BHD-AOT RMs, a strong water–surfactant interaction was detected, while for C_16_TA-AOT, this interaction seemed to be weaker. These results are interesting since a simple change in the cationic component of the catanionic surfactant promotes marked changes in the RM interface, particularly in the water–surfactant interaction. For example, BHD-AOT RMs offer less of an electron donor environment than CTA_16_-AOT. This result may have a tremendous impact and is very useful for their use as nanoreactors [39].

In recent years, a very efficient family of surfactants has been prepared by using alkylimidazolium cations and 1,4-bis-2-ethylhexylsulfosuccinate (as well as its analog 1,4-bis-2-ethylhexylphosphate) as the anion. We have studied this new family, C_n_mim-AOT. C_4_mim-AOT solubility in traditional non-polar solvents used to formulate RMs is reported in Table 3. Remarkably, in the absence of water C_4_mim-AOT is soluble in all aromatic solvents but insoluble in *n*-heptane [66]. However, in the aliphatic solvent, it is possible to prepare RMs at larger [C_4_mim-AOT] than 0.1 M or W_0_ > 0 [67].

**Scheme 3:**
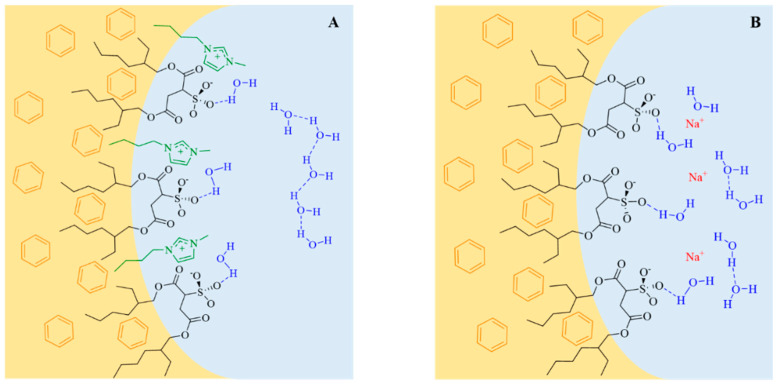
Schematic representation of the location of surfactants ((**A**): 1-butyl-3-methylimidazolium 1,4-bis-2-ethylhexylsulfosuccinate (C_4_mim-AOT). and (**B**): Sodium 1,4-bis-2-ethylhexylsulfosuccinate (Na-AOT) and water molecules at the interface in benzene/surfactant/water RMs. Adapted from Ref. [69] with permission from The Royal Society of Chemistry (2016).

**Figure 1:**
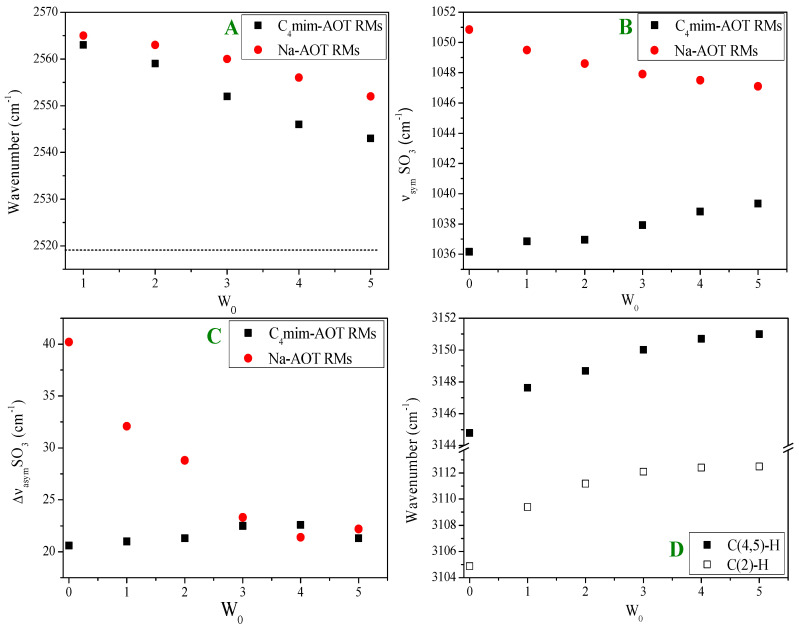
Shift of the FT-IR bands upon increasing W_0_ for (■) 1-butyl-3-methylimidazolium 1,4-bis-2-ethylhexylsulfosuccinate (C_4_mim-AOT) and (●) Sodium 1,4-bis-2-ethylhexylsulfosuccinate (Na-AOT) RMs. (**A**) O-D stretching band (ν_O-D_) for benzene/surfactant/HDO RMs (the value corresponding to neat HDO (----) is included as a reference. (**B**) Splitting of the asymmetric S = O band frequency (Δν_asymSO3_) for chlorobenzene/surfactant/water RMs. (**C**) Shifts of the symmetric S=O stretching band (ν_symSO3_) for chlorobenzene/surfactant/water RMs. (**D**) The shift of the C(2)-H and C(4,5)-H stretching frequencies of C_4_mim^+^ for chlorobenzene/C_4_mim-AOT/water RMs. Adapted from Ref. [69] with permission from The Royal Society of Chemistry (2016).

**Figure 2:**
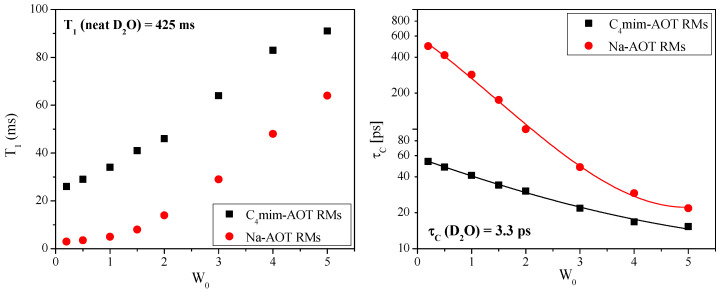
(**left**) *T*_1_ values (in ms) obtained in benzene/surfactant/D_2_O RMs as a function of W_0_. (**right**) Correlation time (in log_10_ scale) of water confined in RMs as a function of W_0_. Adapted from Ref. [69] with permission from The Royal Society of Chemistry (2019).

Evidence of water entrapped by the surfactant creating true RMs was obtained from dynamic light scattering (DLS) experiments because of the droplet size increase with W_0_ with a linear tendency. This feature demonstrates that RMs consist of discrete spherical and non-interacting droplets. The droplet size for C_4_mim-AOT and Na-AOT in benzene was obtained at W_0_ = 5, yielding values of 3.9 nm and 2.9 nm, respectively. This behavior can be explained based on the effective packing parameter, *p* = *v*/*al*_c_, in which *v* and *l_c_* are the volume and the length of the hydrocarbon chain, respectively and *a* is the surfactant head group area [68]. Thus, large reverse micelle sizes relate to small *p* values. Replacement of Na^+^ by C_4_mim^+^ increases the effective area of the ionic-liquid surfactant, *a*, decreasing the packing parameter, *p*, implying that the new counterion, C_4_mim^+^, should be located in a different region compared with sodium as reflected in Scheme 3. This hypothesis was confirmed by FT-IR (Figure 1) and nuclear magnetic resonanse (NMR) experiments (Figure 2) [66,69]. The microenvironment of the RMs can be monitored by FT-IR techniques focusing on the O-D stretching mode of the entrapped water molecules, on the symmetric and asymmetric S=O, carbonyl stretching modes from the AOT moiety, and finally the C-H aromatic stretching mode of C_4_mim^+^. Additionally, NMR has been widely used to investigate the properties of confined water in RMs.

Figure 1A shows the shifts for the ν_O-D_ values corresponding to the O-D stretching band. Values of ν_O-D_ are larger for RMs than neat HDO with Na-AOT also being larger than C_4_mim-AOT. An increase in ν_O-D_ relative to the neat HDO value has been attributed to water molecules interacting with the SO_3_^−^ group disrupting the hydrogen bond network of water. The ν_O-D_ values decrease with increasing W_0_ with different slopes, suggesting a difference in the magnitude of the water-surfactant interaction at the interface.

Figure 1B shows the splitting of the asymmetric S=O stretching band (Δν_asymSO3_). The magnitude of the splitting (Δν_asymSO3_) is indicative of the strength of the perturbation of the SO_3_^-^ by the cation, being larger when the ionic interaction is stronger. In solid Na-AOT, the splitting has a value of 28 cm^−1^ and in benzene, RMs at W_0_ = 0 Δν_asymSO3_ = 42 cm^−1^. In the presence of entrapped water, the magnitude of Δν_asymSO3_ decreases with increasing W_0_ due to weakening of the sodium–sulfonate interaction after hydrogen bond interaction between water and the AOT polar head group. For C_4_mim-AOT RMs at W_0_ = 0, the splitting magnitude Δν_asymSO3_ = 20 cm^−1^ is smaller than for Na-AOT, suggesting a weaker SO_3_^−^-C_4_mim^+^ interaction in comparison with SO_3_^−^-Na^+^.

Figure 1C shows the shifting of the ν_symSO3_ for both surfactants upon increasing W_0_. The shift observed upon increasing W_0_ is opposite in both RMs, and ν_symSO3_ values for W_0_ = 0 are different for C_4_mim-AOT (1036 cm^−1^) and Na-AOT (1050 cm^−1^). Water addition to the Na-AOT RMs causes a shift in the ν_symSO3_ value to lower frequencies. This shift has been attributed to hydrogen bond interactions between the water molecules and the SO_3_^-^ group of AOT. The increase in ν_symSO3_ values with W_0_ for C_4_mim-AOT RMs is attributed to a strengthening of the interactions between the counterion and the polar head group. Herein, as the positive charge is delocalized in the C_4_mim^+^ ion, while it is not in small Na^+^, we expect a stronger interaction between SO_3_^-^ and Na^+^ than with the C_4_mim^+^ cation. The addition of water, which interacts with the SO_3_^-^ group, can reduce the delocalization of negative charge on the O of the SO_3_^-^ group, increasing the frequency of the symmetrical mode. The use of C_4_mim-AOT allows for the analysis of the C-H stretching band (ν_C-H_) of the counterion. Peaks found between 3200 and 3100 cm^−1^ are assigned to the aromatic C-H stretching modes of C(2)-H and C(4,5)-H on the imidazolium cation. These stretching vibration modes can be used to evaluate the cation–anion interaction in RMs since C_4_mim^+^ interacting strongly with anions can diminish its positive charge density with a consequent decrease in the ν_C-H_ values. Both stretching modes shift to higher frequencies with increasing W_0_ (see Figure 1D). A general picture indicates that water, through its free electron pairs, solvates the positive charge of C_4_mim^+^ through the ion–dipole interaction. Thus, when water is added to the C_4_mim-AOT RMs, the initial C_4_mim^+^-SO_3_^−^ interaction at W_0_ = 0 is replaced by water (electron pair)-C_4_mim^+^ and water (hydrogen bond)-SO_3_^−^. Thus, since the water (electron pair)-C_4_mim^+^ interaction is weaker than the electrostatic interaction C_4_mim^+^-SO_3_^-^, the positive charge density on C_4_mim^+^ shifts the C-H vibrations modes to high frequencies. A consequence of this implies that water molecules have less electron donor capacity at the interface than in Na-AOT RMs.

Our group has focused on deuteron NMR relaxometry to directly and unequivocally determine the molecular motion of the entrapped water at the interface of RMs [69] formed with C_4_mim-AOT and Na-AOT. As can be observed in Figure 2-left, at the spin-lattice relaxation time, *T*_1_, values rise as W_0_ increases for both types of RMs, which shows that the water molecules possess more mobility at higher water contents. Note that *T*_1_ values are always much smaller than in bulk water (*T*_1_ = 425 ms), being larger for C_4_mim-AOT than for Na-AOT systems.

Correlation times can be obtained from *T*_1_ (shown in Figure 2-right). The value for bulk water (τ_c_ = 3.3 ps) is included for comparison. The molecular motion of water in the benzene/C_4_mim-AOT/water system is less restricted than that of the water molecules confined in the benzene/Na-AOT/water RMs. Upon increasing W_0_ from 0 to W_0_ = 5, τ_c_ decreases from 54 ps to 15 ps in C_4_mim-AOT RMs instead of the large effect from τ_c_ = 470 ps to τ_c_ = 22 ps observed in Na-AOT systems. This means that water bound to the sulfonate group of AOT in Na-AOT has a very restricted motion concerning the mobility of the interfacial water in the C_4_mim-AOT system. As mentioned above, the location of the C_4_mim^+^ at the interface is crucial to explain the water mobility.

**Scheme 4:**
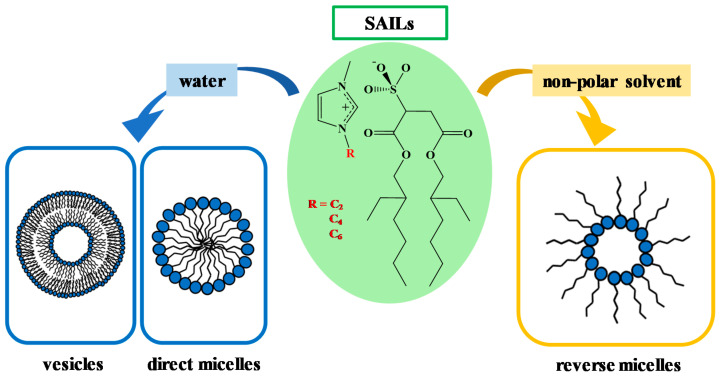
Schematic representation of C_n_mim-AOT self-aggregation in water and non-polar solvents. Adapted with permission from Ref. [70]. Copyright 2020, American Chemical Society.

Recent studies have shown that Na-AOT and C_2_mim-AOT form direct micelles in water, while C_4_mim-AOT and C_6_mim-AOT form vesicles (Scheme 4). Different self-aggregation in water motivated the investigation of RM formation in non-polar solvents as a function of the alkyl chain length of the counterion [70].

The maximum amount of solubilized water (W_0_^max^) in benzene is reported in Table 4. Values of W_0_ depend on the surfactant, indicating that the counterion nature considerably influences the properties of the aggregate. The W_0_^max^ value decreases with increasing hydrocarbon tail length of the imidazolium counterion. The C_6_mim^+^ counterion appears to act as a kind of cosurfactant.

DLS studies reveal the formation of RMs with different droplet sizes (see Table 5). It is interesting to note that the apparent hydrodynamic diameters (d_app_) values of these SAILs RMs are higher than those of Na-AOT in benzene. The d_app_ value increases with the size of the imidazolium cation. Note that based on the critical packing parameter methodology, when *p* decreases, the diameter of the RMs increases. Values of *v* and *l_c_* are similar for both the SAILs and Na-AOT. Substitution of Na^+^ by the C_n_mim^+^ cation can produce an increase in the *a* value because the alkyl tails of the counterions would be close to the chains of AOT, favoring the hydrophobic interactions and producing an increase in d_app_.

In analogy to C_n_mim-AOT, 1-methylimidazolium bis-(2-ethylhexyl)phosphate (imim-DEHP) was prepared and its self-aggregation in water (yielding unilamellar vesicles) and in non-polar solvents (forming RMs) has been studied [71]. Contrary to C_n_mim-AOT, imim-DEHP is soluble in aromatic apolar solvents (benzene or toluene) and aliphatic ones (*n*-heptane) with a strong ability to solubilize water (W_0_^max^ is 39, 15, and 16 in *n*-heptane, benzene, and toluene, respectively). This maximum amount of solubilized water is higher for imim-DEHP than for the same surfactant with sodium counterions (Na-DEHP) as well as C_n_mim-AOT, suggesting that the presence of a protic cationic counterion, which can interact by electrostatic and hydrogen bond interactions with the microenvironment, significantly changes the surfactant behavior. DLS experiments revealed that the hydrodynamic diameter increases linearly with W_0_ in toluene/imim-DEHP/water, with RMs in *n*-heptane being larger than in toluene. Moreover, a clear break in the linear tendency is observed in *n*-heptane that is interpreted as a shape change of the aggregate, with RMs being spherical at W_0_ < 4 and rod-like or ellipsoidal for higher water contents. These differences between aromatic and aliphatic solvents are due to the different degrees of solvent penetration at the interface of the RMs altering the critical packing parameter.

## 3. Biocompatible Solvents as the External Phase in RMs and Microemulsions/Nanoemulsions

Biocompatible W/O microemulsions (or nanoemulsions) and RMs have been proposed for topical, dermal, and transdermal administration of several drugs in the concepts of drug delivery [72,73]. Thus, during the last three decades, a variety of self-organizing systems such as liposomes, niosomes, microemulsions or RMs, organogels, and nanocapsules have been studied as potential systems for drug delivery. When a pharmaceutical formulation needs to deliver an active drug to a definite target organ, it must provide less possible discomfort and side effects to the patient. The concept of drug delivery involves the idea of minimizing the toxic effects of the drugs, improving their solubilization and adsorption, and broadening the modes of administration. Biocompatible RMs adequately prepared may be a solution for most of these aims. Further, these systems have been proven very useful in cosmetic, nutraceutical, and food purposes as promising vectors for hydrophilic, lipophilic, and amphiphilic active molecules [74,75]. Without a doubt, these systems are also considered “the ultimate enzyme microreactors” as reviewed by Mitsou et al. [76].

As the chemical nature of the non-polar component in microemulsion or RMs can be quite diverse, we divided the backgrounds into non-polar solvents and hydrophobic ILs.

### 3.1. Non-Polar Solvents

**Scheme 5:**
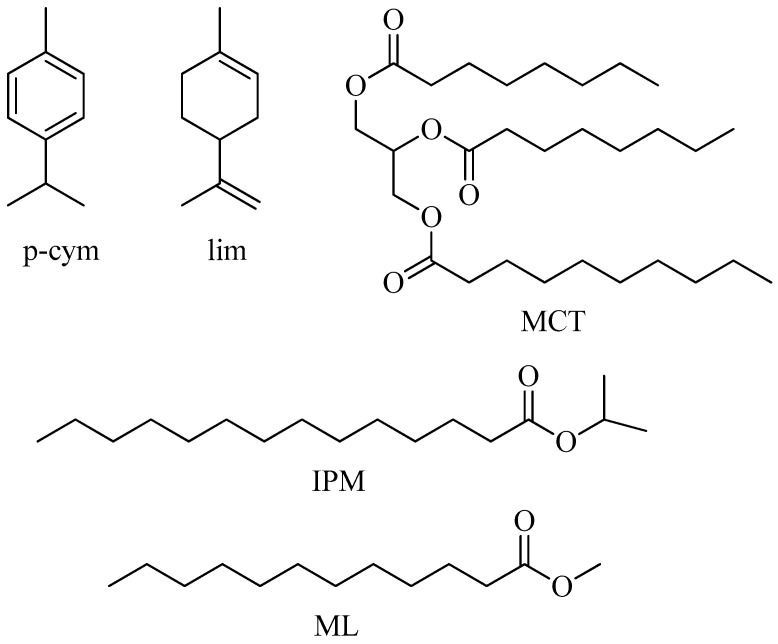
Chemical structure of different non-polar solvents used to formulate biocompatible RMs and microemulsions; p-cymene (p-cym), limonene (lim), medium-chain triglycerides (MCT), isopropyl myristate (IPM), methyl laurate (ML).

One of the fundamental aspects in devising biocompatible RMs is to use non-toxic non-polar solvents as well as biocompatible surfactants and cosurfactants. Regarding the most chosen, biocompatible non-polar solvents are vegetable oils (such as olive oil, corn oil, cottonseed oil, orange oil, clove oil, peppermint oil, eucalyptol oil, and coconut oil), medium-chain triglycerides, and esters of fatty acids (Scheme 5) [72,77].

#### 3.1.1. Vegetable Oils

In the early studies of biocompatible systems, several vegetable oils were employed as the non-polar phase [72]. These oils are usually used with a biocompatible surfactant or mixtures of surfactants and sometimes also cosurfactants. For example, *Eucalyptus* oil was used as the non-polar phase in microemulsions formulated with polyoxyethylene sorbitan monolaurate (Tween 20) as a surfactant and *n*-butanol or cinnamic alcohol as co-surfactants [78]. Cinnamic alcohol was used as a continuous oil phase in microemulsions with Na-AOT or Tween 20 as surfactants and as a nanoreactor to study the fading reaction of the dye crystal violet [79].

Extra virgin olive oil (EVOO) or refined olive oil (ROO) has been used as the non-polar solvent in the formulation of lecithin-based W/O microemulsions which require propanol as a co-surfactant [80]. The phase diagram area of the RMs zone increased considerably by increasing the lecithin-to-propanol weight ratio in both oils. DLS experiments showed that the size of the RMs increased upon water addition. Although the nature of the oil used does not seem to affect the ability of the system to incorporate water, the sizes of the droplets were almost three times smaller when EVOO was used as the non-polar phase. This observation was attributed to the fact that ROO may have endogenous amphiphiles. These RMs were used as nanoreactors to test catalytic activities of two oxidizing enzymes that have been detected in virgin olive oil, namely, tyrosinase and peroxidase, and the activity of a proteolytic enzyme such as trypsin. In all cases, when ROO was used as the oil phase, a reduced catalytic activity was observed. The same RMs composed of olive oil/lecithin/1-propanol/water were used as model biomimetic systems to study the oxidative enzymatic reactions using horseradish peroxidase (HRP) and several substrates with different hydrophilicities [81,82]. Determining the location of the substrate and enzyme, the authors found that the enzyme activity and substrate specificity were significantly affected in olive oil RMs mainly due to the low polarity and the absence of free water in the microenvironment where the enzymes were located and the reactions took place. As observed for other enzymes [83], the activity in these olive oil-based microemulsions was lost with time, depending on the composition and the substrate.

Olive oil/lecithin/1-propanol/water microemulsion has also been used as a biomimetic model to simulate features of the digestion process in the small intestine [84]. The structural and activity studies show the proposed system is quite suitable for an in vitro digestion model. This is because in this system the hydrolytic enzymes, trypsin and alkaline phosphatase, retain the catalytic activity and all other additives tested (o-, m-, and p-coumaric acid, protocatechuic acid, and caffeic acid) are stable and available for the enzymatic reactions, not affecting the structural characteristics of RM droplets.

RMs are based on ROO or sunflower oil (SO), distilled monoglycerides (DMG), and ethanol as a carrier of bioactive nisin [85]. Nisin is a well-known FDA-approved bacteriocin produced by lactic acid bacteria and used in the food industry as a natural bio preservative against food-borne pathogens. The differences in the structural properties of the RMs in oils at constant DMG, ethanol, and water contents demonstrated that SO interacts with the surfactant monolayer, resulting in a slight increase in interfacial flexibility. Accordingly, SAXS measurements for RMs containing ROO, showed a more ordered nanostructure than SO. The inclusion of nisin in these micelles did not produce any alteration, and both systems behaved similarly. The testing of these nisin-loaded RMs showed antimicrobial effects on the tested bacteria.

Edible RMs are formulated with medium-chain triglycerides and ROO as the oil phase, mono, and diglycerides of fatty acids, polysorbate 80 as a surfactant, and water as the dispersed phase. These RMs and SO were used to partially replace hydrogenated kernel palm oil in whipped cream O/W emulsions, yielding healthier and more stable and homogeneous products [86].

Recently, RMs consisting of isopropyl myristate (IPM) or/and EVOO as the continuous phases, Tween 80 and DMG as surfactants, and ultra-pure water were explored [87]. The phase diagrams for EVOO/Tween 80:DMG (1:1)/water were analyzed and compared with the similar RMs which use IPM as the oil phase. Unlike IPM, EVOO has a variety of minor amphiphilic compounds such as free fatty acids and phospholipids that may affect the interfacial membrane of the microemulsion. Moreover, the EVOO-based RMs were used to encapsulate the natural phenolic compound hydroxytyrosol (HT) which has antioxidant, anti-inflammatory, antiproliferative, and cardioprotective properties. The authors used human colon carcinoma Caco-2/TC7 to test in vitro the permeability of HT in comparison with HT aqueous solutions. They found that the absorption of encapsulated HT improved, indicating the importance of the involvement of the amphiphiles in the antioxidant absorption and its entrapment in the mucus layer. Another bioactive antioxidant compound, gallic acid (GA), was also studied in vegetable oil-based RMs [88]. The successful encapsulation of GA in RMs consisting of EVOO and SO as the oil phase, Tween 80 and DMG as biocompatible surfactants, and ultra-pure water was demonstrated. They also added chitosan (CH) as a penetration enhancer. Structural studies showed that the addition of CH did not significantly alter the size or structure as shown by DLS and SAXS. The in vitro tests indicated that GA retains the bioactive properties and that nasal administration is appropriate for the delivery of these compounds.

*R*-(+)-Limonene (lim) has often been used as the oil phase to prepare biocompatible RMs. For example, Papadimitriou et al. [89] used this oil and lecithin as a surfactant and either 1-propanol or 1,2-propanediol as cosurfactants. The composition of the components of the quaternary system was based on the phase diagram experiments. EPR demonstrated that 1-propanol made the interface much more flexible, indicating a less tight packing of lecithin than when 1,2-propanediol was used. The type of co-surfactant also affects the size and amount of maximum water encapsulation as shown by DLS. By electrical conductivity, the percolation threshold was calculated. These RMs were used as nanoreactors in a lipase esterification reaction of octanoic, dodecanoic, and hexadecanoic acids with short-chain alcohols used as co-surfactants. The enzyme efficiency was closely related to the type of co-surfactant, with 1-propanol the most efficient. No ester formation was observed for 1,2-propanediol.

Chatzidaki et al. [90] recently emphasized the need in the food industry to replace high-energy methodologies with sustainable techniques to encapsulate natural additives or preservatives and improve their effectiveness. They encapsulated nisin in essential oil-containing RMs, such as rosemary, thyme, oregano, and dittany essential oils (all of which can enhance the antimicrobial effect of nisin) mixed with ROO as the oil phase and DMG as the surfactant. They studied the RMs using different compositions of the oils and the addition of PG or ethanol by DLS, EPR, and viscosity measurements. They concluded that the essential oils decreased the rigidity of the surfactants’ monolayer and increased the micropolarity of the interface. The interface was more flexible, a fact that can potentially facilitate the diffusion of nisin from the aqueous core to the outer phase which will improve the overall anti-microbial activity. They proved the latter by performing experiments to detect antimicrobial activity in several microorganisms. They also found that nisin-loaded RM, which contains dittany essential oil, had the strongest antibacterial activity.

**Figure 3:**
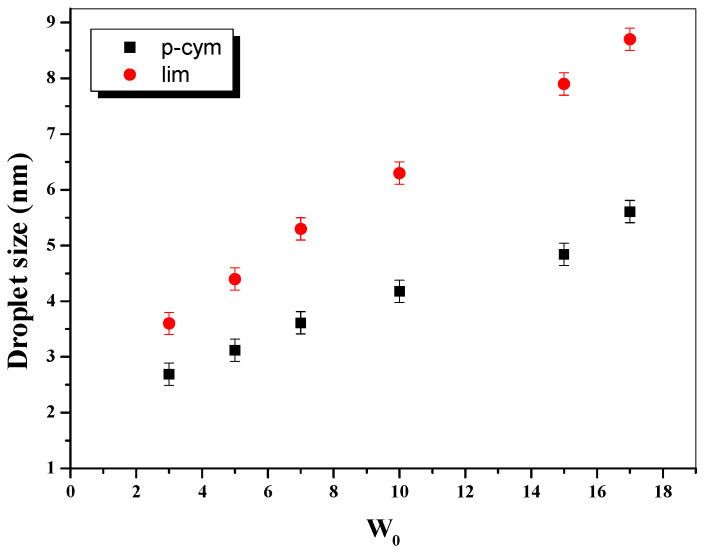
Effect of the water content (W_0_) on the droplet size values in p-cym/Na-AOT/water RMs and lim/Na-AOT/water RMs. T = 25 °C. [Na-AOT] = 0.2 M. Adapted with permission from Ref. [91]. Copyright 2020, American Chemical Society.

Na-AOT has been tested to prepare biocompatible RMs using p-cymene (p-cym) or lim as the oil phase [91]. The authors evaluated the CMC using molecular probes by UV-visible spectroscopy, the size, and effect of water addition by DLS, and the water structure in the droplet by ^1^H NMR. These RMs showed similar features to those observed in Na-AOT RMs formulated in traditional solvents (aromatic and aliphatic) [1] or biocompatible solvents [92]. They are stable systems without co-surfactants and show an increase in size upon water addition, which suggests spherical micelles. The lim-based RMs are larger than the p-cym ones at the same water content, suggesting that p-cym can penetrate the interface more than lim (Figure 3). This leads to a higher aggregation number (N_agg_) for lim/Na-AOT/water RMs compared to p-cym/Na-AOT/water. Furthermore, the ^1^H NMR results showed that the interaction of the water-surfactant at the interface was weaker in p-cym/Na-AOT than in lim/Na-AOT RMs, as expected when the oil penetrates more at the oil–water interface. These biocompatible RMs seem to be a good alternative for a more sustainable system in the multiple application of Na-AOT RMs.

Electrochemical measurements have been proved as a powerful alternative approach to investigate organized media such as RMs [10,93]. In this sense, recently, a new electrochemical method has been applied to a biocompatible W/O stable nanoemulsion [94]. Single-entity electrochemistry (SEE) was used to study highly monodisperse droplets with ~40 nm diameter, consisting of using Pluronic F-127 as a biocompatible surfactant, castor oil as a non-polar medium, and hydrophobic borate compounds as ion exchangers. The SEE technique was combined with nanopipette voltammetry with DLS and TEM to understand mechanistic aspects of the formation of the droplets, the structure of the core, their electrochemical behavior, and the electron transfer reaction oxidation of ferrocene under different conditions. The authors demonstrated that the electrochemical approach provided quantitative criteria for the nanoemulsion investigation as a prerequisite for biomedical application.

An interesting approach has been applied by Kalaitzaki et al. to prepare biocompatible microemulsions based on lemon oil terpenes, polysorbates, water, and glycerol to be used for the formulation of pyrethrins [95]. They prepared lemon oil terpenes/tween 20(tween 80)/water: glycerol(2:1) microemulsion at different compositions. After characterizing the RMs in which the pyrethrins were encapsulated, they were appropriately diluted with water to obtain O/W nanoemulsions to obtain systems suitable to be used successfully as an insecticide.

Rokosik et al. studied the formation of RMs in rapeseed oil using 1,2-dioleoyl-sn-glycero-3-phosphocholine, DOPC, as the surfactant [96,97]. They examined the interactions of sinapic and ferulic acids with the micelles and determined the oil autoxidation process and the antioxidant efficiency of both acids in the oil-containing RMs [97]. Furthermore, they studied the interactions of canolol (4-vinylsyringol) and α-tocopherol (α-T), demonstrating that the DOPC association in RMs influenced the antioxidant efficiency of canolol and α-T.

#### 3.1.2. Medium-Chain Triglycerides

Medium-chain triglycerides (MCT) are also used in biocompatible RMs particularly because they are fully saturated and stable to oxidation [72,73]. Despite the surfactant used, they require the presence of co-surfactants, mostly short-chain alcohols. For example, Garti et al. [98] prepared a food-grade RMs, using several non-ionic biocompatible surfactants, either MCT (the chain-length distribution of the fatty acids was C6:0 ≤ 2%, C8:0 ≤ 50–80%, C10:0 ≤ 18–28%, and C12:0 ≤ 3%) or lim as oil phases and tried ethanol, propylene glycol or glycerol as co-surfactants. They showed by exploring the phase diagrams that the formulation of food-grade microemulsions was difficult to prepare just from three-component systems based on water, oil, and a single surfactant. However, it was possible to formulate these microemulsions by using a suitable non-ionic surfactant and by the addition of polyols and short-chain alcohols.

RMs composed of MCTs as the oil phase, lecithin, and monoglycerides as surfactants, and an aqueous phase were formulated to be used as carriers of natural antioxidants, such as HT and GA [99]. Thus, RMs composed of triglycerides (caprylic/capric) as the oil phase and lecithin and monoglycerides of vegetable fatty acids (DMG 0295) as surfactants were characterized by DLS and EPR. Additionally, a coarse-grained molecular dynamics study based on the experimental composition using the MARTINI force field was performed. In agreement with the experimental data, GA molecules were found to be located in the water core, in contrast to the HT ones, which seem to lie at the surfactant interfacial layer. This difference in the antioxidant′s molecular location was mostly revealed in detail from the computational analysis. Chatzidaki et al. also investigated the role W/O microemulsion upon digestion in the gastrointestinal (GI) tract [100]. They used an MCT, caprylic/capric triglyceride, as the oil phase, lecithin and DMG as surfactants, and 30% *w*/*w* propylene glycol in water as the aqueous phase. They found that that gastric lipolysis in W/O microemulsion was reduced 6- to 9-fold compared with O/W microemulsions. This finding may be a disadvantage for the use of W/O microemulsions as functional foods, but it may have another novel application such as finding a lipid–water interface that could slow down digestion. Similar microemulsions were developed for potential use as functional foods loaded with HT [101]. The authors established the phase diagram and stability of particle size by DLS. Additionally, they used EPR to evaluate the antioxidant capacity of the encapsulated HT. They conclude that these microemulsions have a very good ability to be considered as matrices to encapsulate biologically active hydrophilic molecules.

In the same way, biocompatible topical microemulsions for incorporation and enhanced dermal delivery of sertaconazole nitrate (SN), composed of propylene glycol monocaprylate (C90)/glycereth-7-caprylate:caprate/cosurfactant/water systems, were investigated [102]. The choice of C90 was made considering that if the oil and surfactant have the same length this facilitates the miscibility and subsequent emulsification. They obtained the best formulations using ethanol, isopropyl alcohol, and propylene glycol, as co-surfactants. These formulations had an inverted bicontinuous structure. EPR study suggested the oil phase and the interface on the oil side as the most important places for SN solubilization. The preparations were stable and showed important antifungal applications for damaged skin.

Curcumin is an interesting compound to investigate in drug delivery due to its beneficial effects in carcinogenesis, wound healing, and many inflammatory conditions; however, its solubility is extremely low in both aqueous and non-polar media; consequently, its delivery presents a great challenge. In this sense, recent studies by Nikolic et al. [103,104] in biocompatible microemulsions of Polysorbate 80 and soybean lecithin as surfactants and MCT as the oil phase showed these are effective media for curcumin delivery. They demonstrated that after encapsulation, curcumin interacts with the interface, located in the surfactant layer closer to the lipophilic region. Such a finding implies that curcumin is available for biological reactions, with this system suitable for its delivery.

#### 3.1.3. Lipophilic Fatty Acid esters

The use of lipophilic biocompatible esters has been known since the initial studies of the use of RMs and microemulsions in drug delivery. As a non-toxic lipophilic oil, IPM has been widely used in biologically resembling systems, pharmaceutics, and drug delivery [72].

Hence, Fubini et al. [105] used IPM as an oil phase in the preparation of microemulsions with Na-AOT or egg or soy lecithin as surfactants and *n*-butanol as a co-surfactant. A few years later, Attwood et al. [106] examined the phase diagrams of these W/O microemulsions, particularly the influence of the lecithin/*n*-butanol ratio on the area of existence of the microemulsions. The use of this type of esters was extended to other surfactants [72,73].

**Figure 4:**
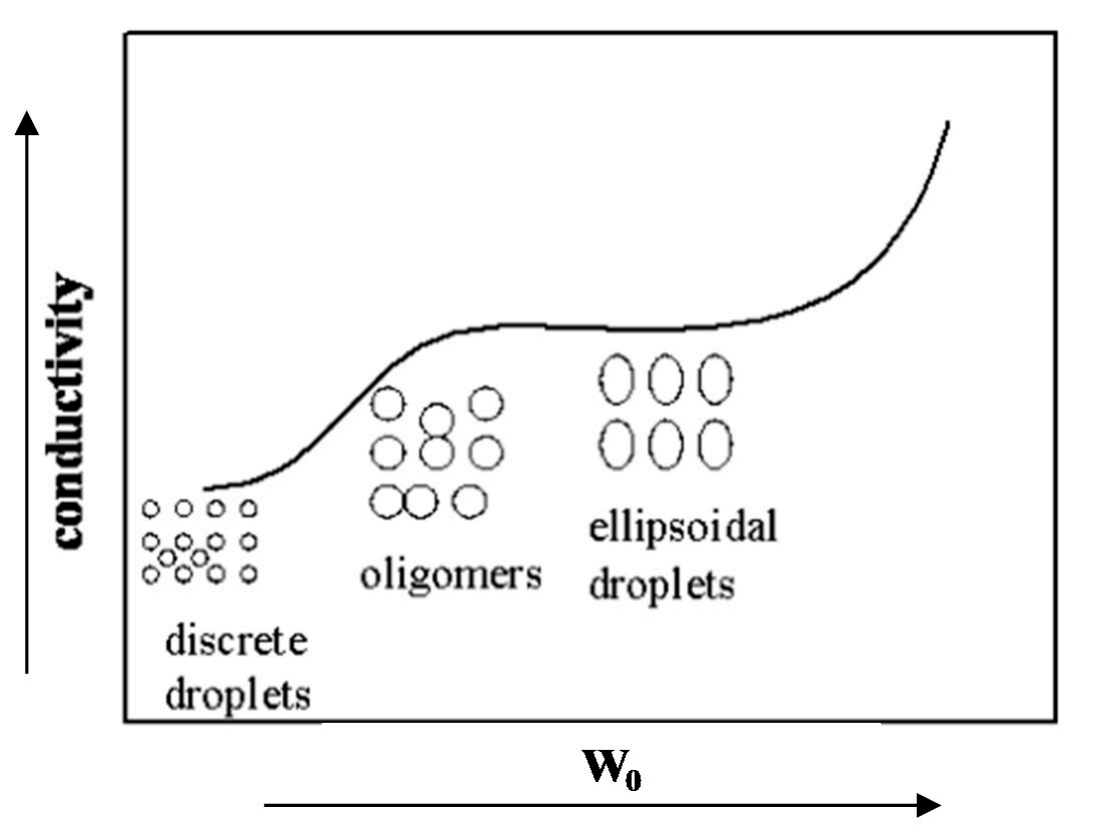
Conductance profile of water/Na-AOT/IPM/Alcohol Systems explored by conductivity measurements. Adapted with permission from Ref. [107]. Copyright 2012, American Chemical Society.

The effect of short-chain alcohols, such as ethanol, propanol, butanol, and pentanol, as co-surfactants on the structure of the water/Na-AOT/IPM RMs, has been systematically investigated [107] using conductivity, DLS, and SAXS techniques. Zhang et al. [107] studied the percolation process induced by water addition and the effect of the alcohol. They found that this phenomenon depends on the type of alcohol, interface flexibility, and interdroplet interactions (Figure 4). It is shown that interfacial flexibility increases when the alcohol chain is shorter and its concentration is increased. Moreover, the droplet attraction is favored by the reduction of the alcohol chain length.

Kundu and Paul [108] studied mixed RMs containing Na-AOT and polyoxyethylene (20) sorbitan trioleate or polyoxyethylene (20) sorbitan monooleate at different compositions formulated in biocompatible oils such as ethyl oleate (EO), isopropyl palmitate (IPP), and IPM. They showed that Na-AOT/polyoxyethylene (20) sorbitan trioleate or Na-AOT/polyoxyethylene (20) sorbitan monooleate/water (aqueous NaCl) formed stable RMs in the three oils. The maximum amount of aqueous NaCl solubilization followed the order IPP < EO < IPM. They discussed why this effect was not expected according to the molar volume of the esters and how the different lengths and branching of the hydrophobic long chain of the esters affected the flexibility or rigidity of the interface, influencing the surface curvature, which consequently affected the solubilization capacity.

IPM was also evaluated as the oil phase in RMs with the mixed surfactant, sodium dodecylsulfate (SDS)/polyoxyethylene (23) lauryl ether (Brij-35) [109]. In this system, 1-pentanol was used as a co-surfactant, and the interfacial compositions and thermodynamic properties were evaluated. The droplet size of these RMs can be adjusted varying not only the water content but also the molar fraction of Brij-35. Liu et al. [110] studied the effect on percolation of the addition of non-ionic surfactants and salts on water/Na-AOT/IPM/alcohol. They used Brij-56, TX-100, Span-20, Span-40, and Span-60 as non-ionic surfactants and salts such as sodium chloride, sodium salicylate, and sodium cholate. Except for sodium chloride, the other salts favored percolation. Taking into account that the addition of non-ionic surfactants increased the interface flexibility, the percolation phenomenon was discussed based on the properties of surfactants and droplet size.

Girardi et al. [92] showed that methyl laurate (ML) and IPM can be used as oil phases in Na-AOT RMs without any co-surfactant. DLS experiments depicted a linear increase in sizes with the water content as expected for the spherical non-interacting droplet. However, at the same W_0_, ML-based RMs were larger than the IPM ones. Further, the N_agg_ was larger for the ML RMs. This fact was explained considering that is possible that IPM, due to its structural properties, can better penetrate the interface, leading to smaller RMs. An interesting feature that should be mentioned is the strong similarity observed when comparing droplet size, the maximum amount of water solubilized, and N_agg_ values between *n*-heptane and ML Na-AOT RMs and benzene and IPM Na-AOT RMs. This could be an alternative when a classical toxic solvent needs to be replaced by a non-toxic solvent for biological applications. More recently, the micropolarity and the hydrogen-bond ability of the interface of these RMs, ML/Na-AOT/water, and IPM/Na-AOT/water were monitored through the use of the solvatochromism of a molecular probe (1-methyl-8-oxyquinolinium betaine, QB) and Fourier transform infrared spectroscopy (FT-IR) [111]. The studies confirmed that due to the dissimilar penetration of these oils into the interfacial region, the micropolarity and the encapsulated water structure were different. Hence, ML-based RMs have a less polar interface than the IPM ones. Moreover, water molecules formed stronger hydrogen bond interactions with the polar head of Na-AOT in ML than in IPM. These results show the importance of the non-polar phase penetration in RMs which allows us to modulate the interface characteristics and consequently future applications.

A study of micellization and RM formation of a cationic gemini surfactant (tetramethylene-1,4-bis(dimethyltetradecylammonium bromide; 14-4-14) has been reported [112]. The authors studied a pseudo ternary system comprised of water, 14-4-14, isopropanol, or n-butanol as cosurfactants and IPM. Further, the effect of the addition of the chemically modified electrolyte sodium carboxymethylcellulose to the water phase was investigated. The systems were studied by conductometry, tensiometry, microcalorimetry, and fluorimetry methods at different temperatures. DLS measurements demonstrated that the droplet size depended on the cosurfactant used; for example, the isopropanol-derived systems were larger than butanol ones, although the sizes also depended on the sodium carboxymethylcellulose addition. These systems were found to have good antimicrobial activity against some Gram-positive and Gram-negative bacteria. The inhibitory effect depended on W_0_, the type of cosurfactant, and the bacterial strains.

With the perspective of their potential pharmaceutical applications, biocompatible microemulsions were prepared as possible novel carriers of theophylline and theobromine [113]. The authors considered three possible microemulsions: (1) IPP/Triton X-100/water + propylene glycol, (2) Miglyol 818/Triton X-100/water + propylene glycol and (3) IPP/Triton X-100 + Span 20/water + propylene glycol. EPR spectroscopy employing the nitroxide spin probe 5-doxylstearic acid, conductivity and DLS were used to characterize interfacial properties of free and loaded systems. The phase diagrams and microemulsions properties depended on the oil and surfactant used but the three microemulsions were suitable for constant and controlled delivery of theophylline and theobromine.

A mixture of MCT and IPM has been used to formulate food-grade microemulsions considering their ability to contain natural food antioxidants such as GA, p-hydroxybenzoic acid, protocatechuic acid, and tyrosol [114]. A typical microemulsion was prepared by mixing IPM and Miglyol 810 (at a weight ratio of 1:1) with a mixture of surfactants/cosurfactants consisting of lecithin/glycerol/ethanol (at a weight ratio of 2:1.7:3.3). To this mixture, the water phase was added up to 2% *w*/*w*. They performed phase diagrams and structural studies using DLS, EPR spectroscopy via a spin probing technique and cryogenic-transmission electron microscopy (cryo-TEM). By DLS, they showed that incorporation of various phenolic compounds in lecithin-based microemulsions resulted in small but significant changes in the apparent hydrodynamic diameter of the aqueous droplets. This was attributed to strong hydrogen bond formation between the phenolic OH and phosphoric groups of the surfactant. By EPR, they observed that phenolic groups may affect the fluidity of the interface, a fact which has to be considered if these microemulsions are used as carriers. By cryo-TEM, they observed the existence of intertwined thread-like micelles.

Guo et al. prepared a biocompatible microemulsion of Tween 60/IPM/ethanol/glycerol/water [115]. In water, these systems undergo a continuous transition from W/O micelles to O/W microemulsion via an ordered bicontinuous structure. They were tested for solubilizing a natural antioxidant β-carotene, whose location in the microemulsion system varies with the structural transition upon dilution of the aqueous phase. Several techniques were employed to characterize the structural transitions upon aqueous phase dilution, including conductivity, viscosity, surface tension, SAXS, and TEM. The authors emphasized the important practical applications of this methodology in water-insoluble nutraceuticals delivery.

Odella et al. also studied biocompatible RMs prepared with mixed surfactants [116]. The RMs were prepared using as surfactant a mixture of Na-AOT and tri-n-octylphosphine oxide (TOPO) surfactants dispersed in ML as the oil phase. The systems were studied by SAXS and ^31^P NMR and by following the solvatochromism of the molecular probe 4-aminophthalimide (4-AP). When compared with pure Na-AOT RMs, TOPO incorporation reduced the droplet size and increased the interfacial fluidity as measured by SAXS and the fluorescence of the molecular probe. ^31^P NMR showed that water was less bound to the mixed interface than in the case of pure Na-AOT RMs. Several properties of these Na-AOT:TOPO mixed RMs are similar to those previously reported in *n*-heptane [117]; however, since they can be generated in a biocompatible solvent such as ML, they are a promissory alternative in the green synthesis of nanoparticles using the RM methodology.

A biocompatible microemulsion was developed using non-ionic surfactants and was investigated as a potential enzyme delivery system for pharmaceutical applications. The system was formulated with IPM/Tween 80/distilled monoglycerides/water/propylene glycol (PG). The system was able to incorporate approximately 3% *w*/*w* aqueous phase containing horseradish peroxidase (HRP) [118]. DLS and EPR techniques showed that the system can incorporate a high range of HRP concentrations. This affects the diameter of the RMs and induces changes in the interface where the enzyme is localized. The increasing PG content increases the hydrodynamic diameter but does not alter the fluidity of the interface. The latter reveals interactions with the inner part of the nanodroplets’ interface. Moreover, the increase of the PG content induced an increased efficiency of HRP to catalyze the oxidation of the model substrate used. The structural study for this formulation in the presence of the enzyme is quite useful in the prediction of the use of biocompatible microemulsions as carriers of therapeutic enzymes and peptides in the treatment of several diseases.

Very recently, Mithila et al. [119] used a formulation created by IPM as oil, Na-AOT as a surfactant, caprylocaproyl polyoxyl-8 glycerides (labrasol) as a co-surfactant, and water as the polar phase. Different ratios of IPM/AOT/labrasol/water were investigated by phase behavior to recognize the concentration range of excipients required to form a monophasic preparation able to encapsulate alendronate (ALD), which is a drug that presents serious gastrointestinal adverse conditions when used in pharmaceutical formulations. In vitro experiments in rat skin revealed significantly enhanced permeation when ALD was incorporated in the microemulsion in comparison with the pure solution. The bioavailability also improved (in vivo studies in Wistar rats), and cell viability assay with human osteoblastic osteosarcoma (MG-63) cells exhibited the positive effects of ALD on cell growth. Moreover, alkaline phosphatase and mineralization studies proved that microemulsion as a carrier retains distinct osteogenic properties of ALD.

Very recently, RM-loaded lipid nanocapsules have been proposed to deliver GA into activated-hepatic stellate cells to suppress hepatic fibrosis progression [120]. The edible RMs were prepared using Labrafil^®^ M 1944 CS (oleoyl polyoxyl-6 glycerides) as oil Kolliphor^®^ RH40 (Macrogolglycerol hydroxystearate, PEG-40 castor oil, and Polyoxyl 40 hydrogenated castor oil) at a surfactant at a ratio of 1:1 (*w*/*w*) where GA was included before preparing the nanocapsules. After characterization of the systems, they showed that this methodology provided efficient delivery of a sufficient amount of GA.

### 3.2. Hydrophobic ILs

As an alternative to traditional non-polar solvents to formulate microemulsion and/or RMs, ILs (hydrophobic) have been used as the external component [121,122,123,124,125,126,127,128,129,130,131,132,133,134,135,136,137,138,139,140,141,142,143,144,145,146]. Particularly, the most used hydrophobic IL is 1-butyl-3-methylimidazolium hexafluorophosphate (bmim-PF_6_) [125,126,127,128,129,130,131,132,134,135,136,137,138,141,142,143,146]. Different surfactants have been employed such as Triton X-100 [125,126,127,128,129,130,131,132,134,135,136,137,140,141,142,143], Tween 20 [138], and Na-AOT [123,124,139,143,146]. Moreover, water was almost exclusively the polar solvent dispersed. Even though these systems have been less explored than those formulated in non-polar solvents, the possibility to create a system where both the internal and external components are polar appears as an interesting medium to be investigated. In this sense, phase behavior [127,130,132,135,138,140,143], sizes [132,134], the aqueous domain existence, and the ability to dissolve ionic compounds [129,132,137,138,143] and enzymes [123,124,141] has been performed in these systems. However, some problems such as large viscosities of the solutions, weak evidence of water structured when it is confined, and the biocompatibility of the ILs used (majority fluorinated derivates) are still a matter of exploration. However, a few examples of possible applications were found [121,122,136,137].

## 4. Biocompatible RMs Formulated with SAILs

The combination of SAILs and biocompatible solvents is a topic that presents an interesting attempt but only a few works are available [147,148,149,150,151,152,153,154].

Rao et al. [147] explored the behavior of RMs formulated by the SAIL N,N-dimethylethanolammonium 1,4-bis(2-ethylhexyl) sulfosuccinate (DA-AOT) dissolved in IPM and the use of other ILs, i.e., 1-hexyl-3-methylimidazolium bis(trifluoromethanesulfonyl)imide (C_6_mim-TF_2_N), as a polar component. They demonstrated a range of thermal stability (278–423 K) of C_6_mim-TF_2_N/DA-AOT/IPM solutions by a phase behavior study, DLS ^1^H NMR, and emission spectroscopy. Thus, the formations of RMs were confirmed by the increase in the size of the droplets as the C_6_mim-TF_2_N content increased. Droplet sizes increased from 7.9 to 91 nm when the C_6_mim-TF_2_N content increased from 0.10 to 0.45. The authors showed a linear tendency of the sizes until the C_6_mim-TF_2_N content was lower than 0.33, and further addition of C_6_mim-TF_2_N caused a deviation from linearity that may be attributed to the increased droplet–droplet interaction. Additionally, the authors demonstrated that the polarity of the polar core inside the organized media was lower in comparison with neat C_6_mim-TF_2_N. Similarly, the same group [148,149] continued the exploration of RMs formulated by SAILs, in this case using the catanionic surfactants C_16_TA-AOT and BHD-AOT dispersed in IPM and entrapping ILs such as N-methyl-N-propylpyrrolidinium bis(trifluoromethanesulfonyl)imide, and N,N,N-trimethyl-N-propylammonium bis-(trifluoromethanesulfonyl)imide.

Wang et al. [150] reported the employment of two ILs, 1-hydroxyethyl-3-methylimidazolium chloride (HOC_2_mim-Cl) and 1-butyl-3-methylimidazolium dodecanesulfate (C_4_mim-C_12_SO_3_), to create a formulation able to dissolve a hydrophilic drug, Dencichine (Den) for topical delivery. The optimal organized system was composed of a mixture of water:HOC_2_mim-Cl (1:1) as a polar component, Tween 80:C_4_mim-C_12_SO_3_ mix (1:1) as surfactants, propylene glycol as a co-surfactant, and IPM as the non-polar phase. This complex mixture (using 50% water:HOC_2_mim-Cl, 20% Tween 80:C_4_mim-C_12_SO_3_, 10% propylene glycol, and 20% of IPM) presented heterogeneous spherical shapes, size of around 47 nm, and zeta potential of −14 mV after Den incorporation. Using this formulation, an interesting increase (~10-fold) in the in vitro skin penetration in comparison with the drug in an aqueous solution was found. Additionally, low cytotoxicity and skin irritation were determined, making this kind of system promising for topical drug administration.

**Figure 5:**
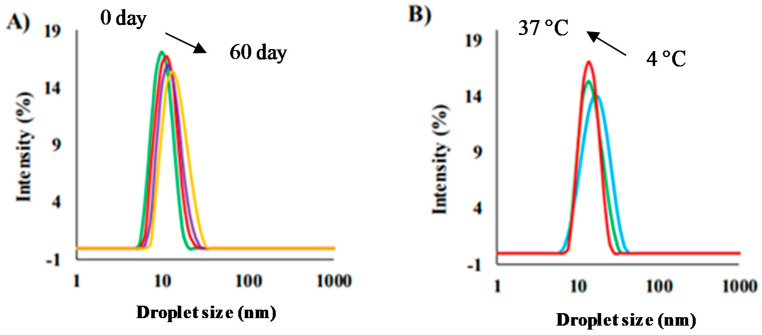
Size distribution of acyclovir-loaded RMs (**A**) effect of storage time at 25 °C and (**B**) effect of storage temperature after two months. RM composition: 3 wt% of choline–propionate, 15 wt% choline–oleate, and Span20 (2:1) in IPM. Adapted from Ref. [151].

Very recently, Goto et al. [151] combined a series of choline-derived (formate, lactate, and propionate) ILs as polar components dispersed in IPM with the help of Choline-Oleate and Span20 as surfactants. After evaluating the phase behavior using different ILs and surfactants/co-surfactants ratios, the droplet size values were monitored by DLS. The diameters changed from 17 to 31 nm by adding more ILs inside. Moreover, the droplet size values increased with increasing choline–oleate and/or decreasing the Span20 content. All cases showed PDI values <0.3, denoting the monodispersity on the systems formed. As a possible application of these systems, the authors chose acyclovir (ACV) as a model sparingly soluble antiviral drug to evaluate. The sizes were explored in the absence and presence of ACV. For example, the size decreased from 31 to 22 nm with increasing ACV amount. The systems were able to dissolve 3–8 mgr of ACV per ml of RMs depending on the composition. Additionally, the systems were evaluated as a function of time and temperature. For example, 3 wt% of choline–propionate, 15 wt% choline–oleate, and Span20 (2:1) in IPM were stable for 60 days (slight increase in size from 22 to 25 nm) and between 4 and 37 °C (Figure 5). Later, an exhaustive investigation about the skin permeability and ACV transdermal and topical delivery was performed, showing an interesting increase in the permeability as a consequence of the choline–oleate as the surfactant. Finally, cytotoxicity studies demonstrated that the systems were non-toxic and safe.

Our group has explored this kind of system, been a pioneer in using a protic IL as the surfactant [152]. To create biocompatible RMs, we combined imim-DEHP as the surfactant, water as the polar phase, and IPM or ML as non-polar solvents. The initial physicochemical characterization started by employing different techniques (DLS, SLS, and FT-IR) to evaluate the capacity of imim-DEHP to formulate true RMs in both non-polar solvents. Our results demonstrated the formation of RMs which responded to the amount of water entrapped, growing in size and producing alterations of the water properties. Interestingly, even though both external solvents are similar in chemical structure, dissimilar behavior between IPM and ML was observed. For example, different amounts of water solubilized, sizes, and aggregation numbers were detected. Additionally, in comparison with previous work [92] using the same biocompatible solvents but Na-AOT as the surfactant, we sensed different water–surfactant interactions at the interface, being stronger in imim-DEHP RMs.

On the other hand, we noted a peculiar dependence on the W_0_ and the type of non-polar solvent if Na-AOT and imim-DEHP were compared. At low W_0,_ the protic surfactant impact on the interfacial properties resulted in the RMs being practically insensitive to the external solvent. In opposition, in Na-AOT/biocompatible RMs [92], the external non-polar solvent strongly influenced the interfacial region, especially at a low amount of water. Thus, the chemical structure of the surfactant (protic vs. aprotic) appears to dominate the RMs. This is important for the applications of RMs.

Very recently [153], we performed a complementary study of both imim-DEHP/biocompatible (IPM and ML) RMs, employing absorption and emission spectroscopy as techniques. Two molecular probes, 3,6-bis (dimethylamino) acridinium bis-(2-ethylhexyl) phosphate (AOBH-DEHP) and N,N,N′,N′-tetramethylethylenediamine copper(II) acetylacetonate tetraphenylborate ([Cu(acac)(tmen)][B(C_6_H_5_)_4_]), were used. Properties such as the CMC values and microviscosity in both systems as a function of the W_0_ were determined. Our results showed that the formation of the RMs is favored when water is present and that the combination of imim-DEHP and the biocompatible solvents generates a very fluid micellar interface. Additionally, a strong interaction between imim^+^ and the water molecules makes the DEHP^−^ moiety a good ligand in comparison to a homogeneous medium. This ability is increased by the water content. Complementary studies varying the non-polar solvents in imim-DEHP RMs demonstrated that the strength of DEHP^-^ to act as a ligand can be modulated by changing the external solvent. Thus, when aliphatic solvents such as *n*-heptane, IPM, and ML were used, a greater increase in the strength of DEHP^-^ as a ligand was obtained, while in aromatic solvents, the increase in strength as a ligand was less marked. These facts were interpreted as a consequence of the larger penetration of aromatic solvents into the micellar interface, which interferes in the imim^+^-water interaction releasing to DEHP^-^. These findings are promising in applications of these systems to be used in metal extraction.

SAILs derived from AOT such as C_4_mim-AOT were also investigated in biocompatible solvents. IPM and ML non-polar solvents and water were employed as external and polar phases, respectively [154]. By using DLS and ^1^H NMR as techniques, the effect to replace the non-polar solvent *n*-heptane with IPM and ML was explored and compared with the Na-AOT RMs in the same solvents. The results indicated that in comparison to *n*-heptane, IPM and ML penetrate more to the micellar interface. Thus, the C_4_mim^+^-AOT^-^ interaction is influenced by the closeness of the ester group of IPM or ML to the interfacial region. Additionally, this fact generates similar behavior between IPM and ML in C_4_mim-AOT RMs, because the micellar interface properties are strongly governed by the C_4_mim^+^ counterion more than by the type of external solvent used. However, a dissimilar penetration of each biocompatible solvent makes the interfacial properties of Na-AOT RMs different.

**Figure 6:**
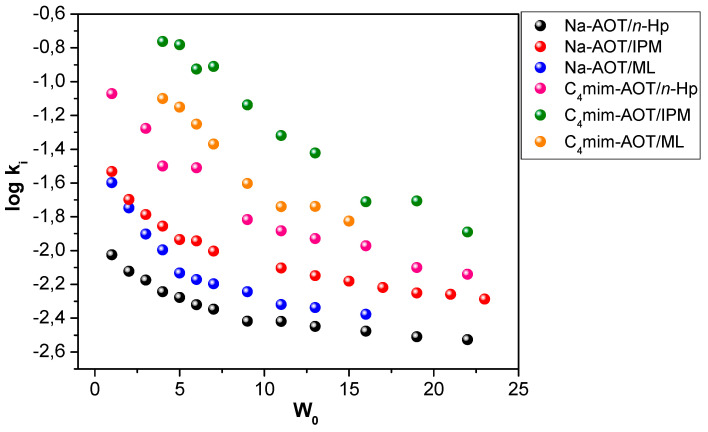
Log ki as a function of the water content (W_0_) for the CF_3_ hydrolysis reaction in Na-AOT and C_4_mim-AOT RMs using n-Hp, IPM, and ML as nonpolar external solvents. Adapted with permission from Ref. [155]. Copyright 2020, American Chemical Society.

Very recently, Dib et al. [155] explored two hydrolysis reactions as a probe to investigate the properties of RMs formed by C_4_mim-AOT using as the external non-polar solvents, *n*-heptane, IPM, and ML. The substrates chosen were 4-methoxybenzoyl chloride (OMe) and 4-(trifluoromethyl)benzoyl chloride (CF_3_). Interestingly, the results indicate that the replacement of the Na^+^ counterion by C_4_mim^+^ in AOT RMs alters the rates of reactions carried out in them and produces changes in the reaction mechanism. In C_4_mim-AOT RMs, the C_4_mim^+^ cation influences the reaction intermediates′ stability and, therefore, the reaction rates and mechanism. Further, the results indicate that when IPM is used as an external solvent instead of ML or *n*-heptane, interfacial water molecules have larger nucleophilicity due to the higher interface penetration of IPM (Figure 6).

**Scheme 6:**
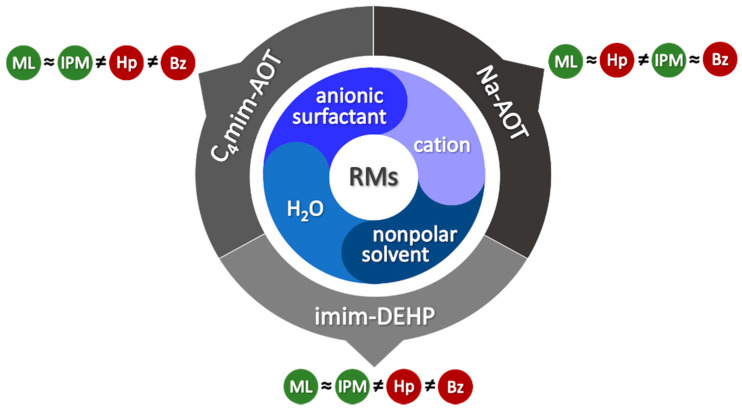
Schematic representation of the effect of changing the RM components on the external solvent behavior. (Hp: *n*-heptane, Bz: benzene, IPM: isopropyl myristate, ML: methyl laurate).

In summary, when new RMs are formed, several aspects have to be considered such as external solvent penetration, water content, and the surfactant structure (including the counterion) to understand the observed properties. Thus, by changing the components, different effects are observed and predictability is not possible. For example, at low W_0_ the interfacial water–surfactant interaction dominates the non-polar solvent penetration more than the chemical structure of the surfactant (Scheme 6). A different situation is observed at high water content, where the surfactant structure appears more determining than the non-polar external solvent.

Finally, it is important to mention that the toxicity of ILs, in general, is still under evaluation. Considering the variety of chemical structures that they present, several experiments and tests are needed to classify them simply as toxic or safe [13]. It is crucial to understand their biodegradability and metabolites to infer their impact on the environment. Two strategies can be employed for specific applications, to eliminate their toxicity by changing their chemical structure and/or minimize the amount used to maximize their properties. The latter strategy is what we want to highlight in this review. Irrespective, many of them are used in pharmaceutical products [12].

## 5. Conclusions and Future Perspectives

In this review, we present works that demonstrate how two types of sustainable compounds such as biocompatible solvents and SAILs can be combined to generate novel organized systems such as RMs. Further, the variety of known preparations of biocompatible microemulsions with these types of solvents has been reviewed. Considering these backgrounds, as can be seen, the properties of the RMs can be greatly modified by changes in the type of surfactant and external solvents, and all the modifications generate novel systems with dissimilar properties. These unique properties cannot be anticipated or extrapolated, and deep analysis is always required. Finally, the works described provide valuable information about the use of biocompatible RMs, making them a green and promising alternative toward efficient and sustainable chemistry. In this sense, we believe that these findings could stimulate other scientists to use these kinds of RMs in diverse applications such as material synthesis or biological transformations, in drug delivery, cosmetics, or food science.

## Figures and Tables

**Table 1:** Chemical structure of the most common surface-active ionic liquids (SAILs).

Surfactant	Ref.
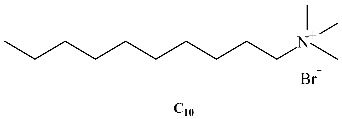	CMC = 6.1 × 10^−2^ M [37]
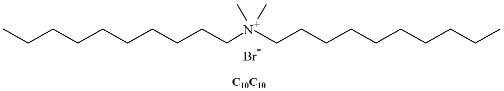	CMC = 1.85 × 10^−3^ M [37]
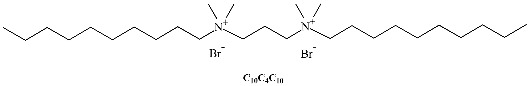	CMC = 8.78 × 10^−3^ M [37]
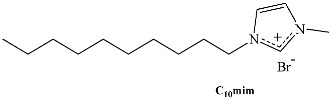	CMC = 2.0 × 10^−2^ M [37]
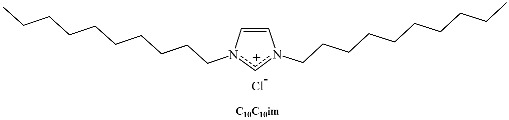	CMC = 4.5 × 10^−4^ M [37]
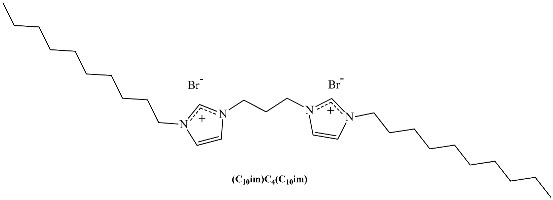	CMC = 2.0 × 10^−3^ M [37]
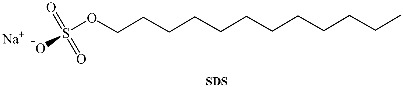	CMC = 8.0 × 10^−3^ M [38]
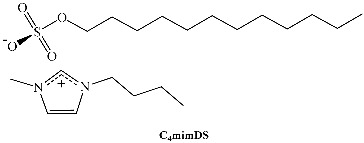	CMC = 2.30 × 10^−3^ M [38]
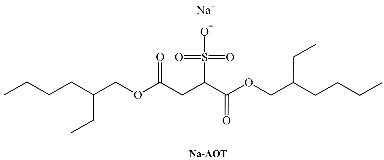	CMC = 2.88 × 10^−3^ M [38]
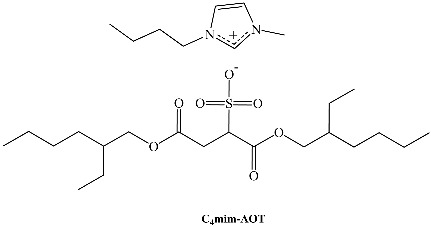	CMC = 1.78 × 10^−3^ M [38]
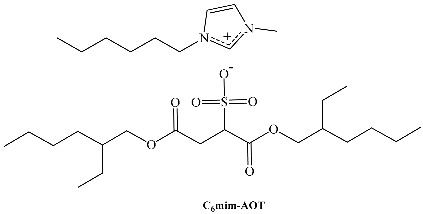	CMC = 7.7 × 10^−4^ M
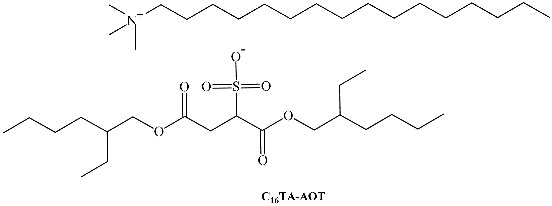	[39,40]
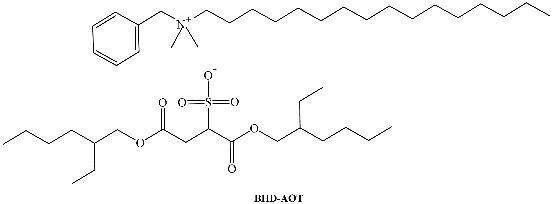	[39,40]
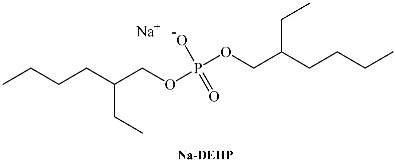	CMC = 1.59 × 10^−2^ M [41]
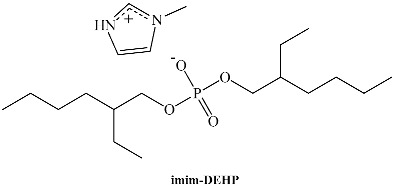	[42]

**Table 2:** Reverse micelles (RMs) formulated with (ionic liquids) ILs and their main characteristics.

IL	Non-Polar Phase	Surfactant/Co-Surfactant	RM Type	Refenence
[C_16_mim][Br]	*p*-xylene		Hexagonal and lamellar lyotropic liquid crystals	[56]
[C_16_mim][Cl]	1-decanol		Hexagonal and lamellar lyotropic liquid crystals	[57]
[C_14_mim][Br]	*p*-xylene		Hexagonal and lamellar lyotropic liquid crystals	[58]
[NH_2_ebim][BF_4_]	cyclohexane	TX-100/1-butanol	W/O, BC, O/W	[59]
[C_2_mim][Cl]	isooctane	Na-AOT	W/O	[60]
[C_4_mim][Cl]	isooctane	Na-AOT	W/O	[60]
[C_8_mim][Cl]	isooctane	Na-AOT	W/O	[60]
[C_16_mim][Cl]	*n*-heptane	Brij-58/ 1-pentanol	O/W	[61]
[C_4_mim][BF_4_]	*n*-heptane	DODMAC:SDS/n-butanol	W/O, BC, O/W	[62]
[C_12_mim][(^i^C_8_)_2_PO_2_]	*n*-heptane	-----	W/O, BC, O/W	[63]

**Table 3:** Maximum amount of water solubilized [66] (W_0_^max^) in different systems at [surfactant] = 0.1 M. T = 25.0 °C; 1-butyl-3-methylimidazolium 1,4-bis-2-ethylhexylsulfosuccinate (C_4_mim-AOT). Sodium 1,4-bis-2-ethylhexylsulfosuccinate (Na-AOT).

	W_0_^max^	
Non-Polar Solvent	C_4_mim-AOT	Na-AOT
*n*-heptane		60
benzene	5.0	12
chlorobenzene	5.4	8
toluene	5.1	10

**Table 4:** The maximum amount of solubilized water (W_0_^max^) in different systems prepared in benzene at [surfactant] = 0.1 M. T = 25.0 °C.

	Surfactant			
	Na-AOT	C_2_mim-AOT	C_4_mim-AOT	C_6_mim-AOT
W_0_^max^	12	11	5	2

**Table 5:** Apparent hydrodynamic diameters (d_app_) and polydispersity indices (PDIs) for benzene/surfactant/water RMs at W_0_ = 5.

Surfactant	dapp (nm)	PDI
Na-AOT	2.9 ± 0.3	0.05
C_2_mim-AOT	3.5 ± 0.2	0.04
C_4_mim-AOT	3.9 ± 0.2	0.03
C_6_mim-AOT ^a^	5.3 ± 0.1	0.02

^a^ Value for benzene/C_6_mim-AOT/water was obtained at W_0_ = 2.

## Data Availability

Not applicable.

## Glossary

Cu(acac)(tmen)][B(C_6_H_5_)_4_]	N,N,N′,N′-tetramethylethylenediamine copper(II)acetylacetonate tetraphenylborate.
ACV	acyclovir
ALD	alendronate
AOBH-DEHP	3,6-bis (dimethylamino) acridinium bis-2-ethylhexylphosphate
AOT	1,4-bis-2-ethylhexylsulfosuccinate
BHD-AOT	benzyl-n-hexadecyldimethylammonium 1,4-bis-2-ethylhexylsulfosuccinate
BHDC	benzyl-n-hexadecyldimethylammonium chloride
C_4_mim-AOT	1-butyl-3-methylimidazolium 1,4-bis-2-ethylhexylsulfosuccinate
Brij-35	polyoxyethylene (23) lauryl ether
Capryol™ 90, C90	propylene glycol monocaprylate
CH	chitosan
CMC	critical micelle concentration
C_16_TA-AOT	cetyltrimethylammonium 1,4-bis(2-ethylhexyl)sulfosuccinate
Cryo-TEM	cryogenic-transmission electron microscopy
DA-AOT	N,N-dimethylethanolammonium 1,4-bis-2-ethylhexylsulfosuccinate
DLS	Dynamic Light Scattering
DMG	distilled monoglycerides
DOSY	Diffusion Ordered Spectroscopy
DSC	differential scanning calorimetry
E471	Mono and diglycerides of fatty acids
Emanon EV-E, EV	glycereth-7-caprylate/caprate
EO	ethyl oleate
EPR	electron paramagnetic resonance
EVOO	Extra virgin olive oil
FTIR	Fourier transform infrared spectroscopy
GA	gallic acid
HDEHP	bis-(2- ethylhexyl)phosphoric acid
C_6_mim-AOT	1-hexyl-3-methylimidazolium 1,4-bis-2-ethylhexylsulfosuccinate
C_6_mim-TF_2_N	1-hexyl-3-methylimidazolium bis(trifluoromethanesulfonyl)imide
HOC_2_mim-Cl	1-hydroxyethyl-3-methylimidazolium chloride
HRP	horseradish peroxidase
HT	hydroxytyrosol
ILs	Ionic Liquids
imim-DEHP	1-methylimidazolium bis-2-ethylhexylphosphate
IPM	Isopropyl Myristate
IPP	isopropyl palmitate
Lim	(R)-(+) limonene
MCT	medium chain triglycerides
Miglyoil^®^810	caprylic/capric triglyceride
Miglyol^®^ 818	caprylic/capric/linoleic triglyceride
ML	Methyl Laurate
Na-AOT	Sodium 1,4-bis-2-ethylhexylsulfosuccinate
Na-DEHP	sodium bis-2-ethylhexylphosphate
N_agg_	aggregation number
O/W	oil in water
p-cym	p-cymene
PG	1,2-propanediol
RMs	Reverse Micelles
ROO	refined olive oil
SAIL	surface active ionic liquid
SAXS	Small Angle X-Ray Scattering
SDS	sodium dodecylsulfate
SEE	single-entity electrochemistry
SO	sunflower oil
Span 20	sorbitan monolaurate
TEM	transmission electron microscopy
TOPO	tri-n-octylphosphine oxide
Triton X-100	1-Octylphenoxy-polyethoxyethanol
Tween 20	Polyoxyethylene sorbitan monolaurate
Tween 60	polyoxyethylene (20) sorbitan monostearate
Tween 80	Polyoxyethylene sorbitan monooleate
W/O	water- in oil
W_0_ = [H_2_O]/[surfactant]	

## References

[1] Correa N.M., Silber J.J., Riter R.E., Levinger N.E. (2012). Nonaqueous Polar Solvents in Reverse Micelle Systems. Chem. Rev..

[2] Silber J.J., Biasutti A., Abuin E., Lissi E. (1999). Interactions of small molecules with reverse micelles. Adv. Colloid Interface Sci..

[3] Glatter O., Salentinig S. (2020). Inverting structures: From micelles via emulsions to internally self-assembled water and oil continuous nanocarriers. Curr. Opin. Colloid Interface Sci..

[4] Wong F.W.F., Ariff A.B., Stuckey D.C. (2018). Downstream protein separation by surfactant precipitation: A review. Crit. Rev. Biotechnol..

[5] Danielsson I., Lindman B. (1981). The definition of microemulsion. Colloids Surf..

[6] Rehman F.U., Shah K.U., Shah S.U., Khan I.U., Khan G.M., Khan A. (2017). From nanoemulsions to self-nanoemulsions, with recent advances in self-nanoemulsifying drug delivery systems (SNEDDS). Expert Opin. Drug Deliv..

[7] Simonazzi A., Cid A.G., Villegas M., Romero A.I., Palma S.D., Bermúdez J.M., Grumezescu A.M. (2018). Chapter 3—Nanotechnology applications in drug controlled release. Drug Targeting and Stimuli Sensitive Drug Delivery Systems.

[8] Choudhury H., Pandey M., Gorain B., Chatterjee B., Madheswaran T., Md S., Mak K.K., Tambuwala M., Chourasia M.K., Kesharwani P., Kesharwani P. (2019). Chapter 9—Nanoemulsions as Effective Carriers for the Treatment of Lung Cancer. Nanotechnology-Based Targeted Drug Delivery Systems for Lung Cancer.

[9] Kumar H., Kumar V. (2018). Ultrasonication assisted formation and stability of water-in-oil nanoemulsions: Optimization and ternary diagram analysis. Ultrason. Sonochem..

[10] Florez Tabares J.S., Correa N.M., Silber J.J., Sereno L.E., Molina P.G. (2015). Droplet-droplet interactions investigated using a combination of electrochemical and dynamic light scattering techniques. the case of water/BHDC/benzene:n-heptane system. Soft Matter.

[11] Orellano M.S., Longo G.S., Porporatto C., Correa N.M., Falcone R.D. (2020). Role of micellar interface in the synthesis of chitosan nanoparticles formulated by reverse micellar method. Colloids Surf. A Physicochem. Eng. Asp..

[12] Md Moshikur R., Chowdhury M.R., Moniruzzaman M., Goto M. (2020). Biocompatible ionic liquids and their applications in pharmaceutics. Green Chem..

[13] Magina S., Barros-Timmons A., Ventura S.P.M., Evtuguin D.V. (2021). Evaluating the hazardous impact of ionic liquids—Challenges and opportunities. J. Hazard. Mater..

[14] Welton T. (2006). All solutions have a solvent. Green Chem..

[15] Welton T. (2018). Ionic liquids: A brief history. Biophys. Rev..

[16] Welton T. (1999). Room-Temperature Ionic Liquids. Solvents for Synthesis and Catalysis. Chem. Rev..

[17] Wasserscheid P., Welton T., Wasserscheid P., Welton T. (2007). Ionic Liquids in Synthesis.

[18] Welton T. (2004). Ionic liquids in catalysis. Coord. Chem. Rev..

[19] Hallett J.P., Welton T. (2011). Room-temperature ionic liquids: Solvents for synthesis and catalysis. 2. Chem. Rev..

[20] Nasirpour N., Mohammadpourfard M., Zeinali Heris S. (2020). Ionic liquids: Promising compounds for sustainable chemical processes and applications. Chem. Eng. Res. Des..

[21] Falcone R.D., Correa N.M., Silber J.J. (2020). Amphiphilic ionic liquids as sustainable components to formulate promising vesicles to be used in nanomedicine. Curr. Opin. Green Sustain. Chem..

[22] Pacheco-Fernández I., González-Hernández P., Pino V., Ayala J.H., Afonso A.M. (2018). CHAPTER 3 Ionic Liquid-based Surfactants: A Step Forward. Ionic Liquid Devices.

[23] Paul B.K., Moulik S.P., Paul B.K., Moulik S.P. (2015). Ionic Liquid-Based Surfactant Science.

[24] Blesic M., Marques M.H., Plechkova N.V., Seddon K.R., Rebelo L.P.N., Lopes A. (2007). Self-aggregation of ionic liquids: Micelle formation in aqueous solution. Green Chem..

[25] Bhadani A., Misono T., Singh S., Sakai K., Sakai H., Abe M. (2016). Structural diversity, physicochemical properties and application of imidazolium surfactants: Recent advances. Adv. Colloid Interface Sci..

[26] Cao H., Hu Y., Xu W., Wang Y., Guo X. (2020). Recent progress in the assembly behavior of imidazolium-based ionic liquid surfactants. J. Mol. Liq..

[27] Geng F., Liu J., Zheng L., Yu L., Li Z., Li G., Tung C. (2010). Micelle Formation of Long-Chain Imidazolium Ionic Liquids in Aqueous Solution Measured by Isothermal Titration Microcalorimetry. J. Chem. Eng. Data.

[28] Qiu Z., Texter J. (2008). Ionic liquids in microemulsions. Curr. Opin. Colloid Interface Sci..

[29] Friesen S., Buchecker T., Cognigni A., Bica K., Buchner R. (2017). Hydration and Counterion Binding of [C12MIM] Micelles. Langmuir.

[30] Vasiloiu M., Rainer D., Gaertner P., Reichel C., Schröder C., Bica K. (2013). Basic chiral ionic liquids: A novel strategy for acid-free organocatalysis. Catal. Today.

[31] Cognigni A., Kampichler S., Bica K. (2017). Surface-active ionic liquids in catalysis: Impact of structure and concentration on the aerobic oxidation of octanol in water. J. Colloid Interface Sci..

[32] Taskin M., Cognigni A., Zirbs R., Reimhult E., Bica K. (2017). Surface-active ionic liquids for palladium-catalysed cross coupling in water: Effect of ionic liquid concentration on the catalytically active species. RSC Adv..

[33] Ding Y.-S., Zha M., Zhang J., Wang S.-S. (2007). Synthesis, characterization and properties of geminal imidazolium ionic liquids. Colloids Surf. A Physicochem. Eng. Asp..

[34] Baltazar Q.Q., Chandawalla J., Sawyer K., Anderson J.L. (2007). Interfacial and micellar properties of imidazolium-based monocationic and dicationic ionic liquids. Colloids Surf. A Physicochem. Eng. Asp..

[35] Pal A., Datta S., Aswal V.K., Bhattacharya S. (2012). Small-Angle Neutron-Scattering Studies of Mixed Micellar Structures Made of Dimeric Surfactants Having Imidazolium and Ammonium Headgroups. J. Phys. Chem. B.

[36] Zana R. (1980). Ionization of cationic micelles: Effect of the detergent structure. J. Colloid Interface Sci..

[37] Figueira-González M., Francisco V., García-Río L., Marques E.F., Parajó M., Rodríguez-Dafonte P. (2013). Self-Aggregation Properties of Ionic Liquid 1,3-Didecyl-2-methylimidazolium Chloride in Aqueous Solution: From Spheres to Cylinders to Bilayers. J. Phys. Chem. B.

[38] Brown P., Butts C.P., Eastoe J., Fermin D., Grillo I., Lee H.C., Parker D., Plana D., Richardson R.M. (2012). Anionic surfactant ionic liquids with 1-Butyl-3-methyl-imidazolium cations: Characterization and application. Langmuir.

[39] Villa C.C., Correa N.M., Silber J.J., Falcone R.D. (2019). Catanionic Reverse Micelles as an Optimal Microenvironment to Alter the Water Electron Donor Capacity in a S_N_2 Reaction. J. Org. Chem..

[40] Villa C.C., Silber J.J., Correa N.M., Falcone R.D. (2014). Effect of the Cationic Surfactant Moiety on the Structure of Water Entrapped in Two Catanionic Reverse Micelles Created from Ionic Liquid-Like Surfactants. ChemPhysChem.

[41] Cheng N., Ma X., Sheng X., Wang T., Wang R., Jiao J., Yu L. (2014). Aggregation behavior of anionic surface active ionic liquids with double hydrocarbon chains in aqueous solution: Experimental and theoretical investigations. Colloids Surf. A Physicochem. Eng. Asp..

[42] Andrada H.E., Silva O.F., Morales G.M., Correa N.M., Falcone R.D. (2020). Spontaneous formation of unilamellar vesicles based on the surfactant 1-methylimidazolium bis-(2-ethylhexyl) phosphate, evaluated as a function of pH and in saline solution. Colloids Surf. A Physicochem. Eng. Asp..

[43] Rosen M.J. (2004). Surfactants and Interfacial Phenomena.

[44] Nandwani S.K., Malek N.I., Lad V.N., Chakraborty M., Gupta S. (2017). Study on interfacial properties of Imidazolium ionic liquids as surfactant and their application in enhanced oil recovery. Colloids Surf. A Physicochem. Eng. Asp..

[45] Pillai P., Pal N., Mandal A. (2017). Synthesis, Characterization, Surface Properties and Micellization Behaviour of Imidazolium-based Ionic Liquids. J. Surfactants Deterg..

[46] Yousefi M., Naseri A., Abdouss M., Miran Beigi A.A. (2017). Synthesis and characterization of eight hydrophilic imidazolium-based ionic liquids and their application on enhanced oil recovery. J. Mol. Liq..

[47] Rodríguez-Palmeiro I., Rodríguez-Escontrela I., Rodríguez O., Arce A., Soto A. (2015). Characterization and interfacial properties of the surfactant ionic liquid 1-dodecyl-3-methyl imidazolium acetate for enhanced oil recovery. RSC Adv..

[48] Wang H., Wang J., Zhang S., Xuan X. (2008). Structural Effects of Anions and Cations on the Aggregation Behavior of Ionic Liquids in Aqueous Solutions. J. Phys. Chem. B.

[49] Kamboj R., Bharmoria P., Chauhan V., Singh G., Kumar A., Singh S., Kang T.S. (2014). Effect of cationic head group on micellization behavior of new amide-functionalized surface active ionic liquids. Phys. Chem. Chem. Phys..

[50] Ao M.Q., Xu G.Y., Zhu Y.Y., Bai Y. (2008). Synthesis and properties of ionic liquid-type Gemini imidazolium surfactants. J. Colloid Interface Sci..

[51] Rodríguez-Escontrela I., Rodríguez-Palmeiro I., Rodríguez O., Arce A., Soto A. (2015). Phase behavior of the surfactant ionic liquid trihexyltetradecylphosphonium bis(2,4,4-trimethylpentyl)phosphinate with water and dodecane. Colloids Surf. A Physicochem. Eng. Asp..

[52] Sakthivel S., Gardas R.L., Sangwai J.S. (2016). Effect of Alkyl Ammonium Ionic Liquids on the Interfacial Tension of the Crude Oil–Water System and Their Use for the Enhanced Oil Recovery Using Ionic Liquid-Polymer Flooding. Energy Fuels.

[53] Manshad A.K., Rezaei M., Moradi S., Nowrouzi I., Mohammadi A.H. (2017). Wettability alteration and interfacial tension (IFT) reduction in enhanced oil recovery (EOR) process by ionic liquid flooding. J. Mol. Liq..

[54] Nandwani S.K., Malek N.I., Chakraborty M., Gupta S. (2020). Insight into the Application of Surface-Active Ionic Liquids in Surfactant Based Enhanced Oil Recovery Processes–A Guide Leading to Research Advances. Energy Fuels.

[55] Hejazifar M., Lanaridi O., Bica-Schröder K. (2020). Ionic liquid based microemulsions: A review. J. Mol. Liq..

[56] Zhang J., Dong B., Zheng L., Li N., Li X. (2008). Lyotropic liquid crystalline phases formed in ternary mixtures of 1-cetyl-3-methylimidazolium bromide/p-xylene/water: A SAXS, POM, and rheology study. J. Colloid Interface Sci..

[57] Zhang G., Chen X., Xie Y., Zhao Y., Qiu H. (2007). Lyotropic liquid crystalline phases in a ternary system of 1-hexadecyl-3-methylimidazolium chloride/1-decanol/water. J. Colloid Interface Sci..

[58] Li X.-W., Zhang J., Zheng L.-Q., Chen B., Wu L.-Z., Lv F.-F., Dong B., Tung C.-H. (2009). Microemulsions of N-Alkylimidazolium Ionic Liquid and Their Performance as Microreactors for the Photocycloaddition of 9-Substituted Anthracenes. Langmuir.

[59] Liu H.J., Li B., Yao H., Pan Y., Wang Y. (2017). A new type of microemulsion containing task-special ionic liquid [NH2ebim][BF4]. J. Dispers. Sci. Technol..

[60] Wei J., Su B., Yang J., Xing H., Bao Z., Yang Y., Ren Q. (2011). Water solubilization capacity and volume-induced percolation of sodium bis(2-ethylhexyl)sulfosuccinate microemulsions in the presence of 1-alkyl-3-methylimidazolium chloride ionic liquids. J. Chem. Eng. Data.

[61] Kundu K., Bardhan S., Ghosh S., Saha S.K., Paul B.K. (2016). Formation of Oil/Water Interface by Mixed Surface Active Ionic Liquid-Ethoxylated Alkyl Ether: Energetics, Microstructures, Solvation Dynamics, and Antimicrobial Activity. ChemistrySelect.

[62] Liu L., Bauduin P., Zemb T., Eastoe J., Hao J. (2009). Ionic Liquid Tunes Microemulsion Curvature. Langmuir.

[63] Hejazifar M., Earle M., Seddon K.R., Weber S., Zirbs R., Bica K. (2016). Ionic Liquid-Based Microemulsions in Catalysis. J. Org. Chem..

[64] Villa C.C., Silber J.J., Falcone R.D., Correa N.M. (2017). Subtleties of catanionic surfactant reverse micelle assemblies revealed by a fluorescent molecular probe. Methods Appl. Fluoresc..

[65] Villa C.C., Moyano F., Ceolin M., Silber J.J., Falcone R.D., Correa N.M. (2012). A unique ionic liquid with amphiphilic properties that can form reverse micelles and spontaneous unilamellar vesicles. Chem. A Eur. J..

[66] Lépori C.M.O., Correa N.M., Silber J.J., Falcone R.D. (2016). How the cation 1-butyl-3-methylimidazolium impacts the interaction between the entrapped water and the reverse micelle interface created with an ionic liquid-like surfactant. Soft Matter.

[67] Dib N., Falcone R.D., Acuña A., García-Río L. (2019). Characterization of Reverse Micelles Formulated with the Ionic-Liquid-like Surfactant Bmim-AOT and Comparison with the Traditional Na-AOT: Dynamic Light Scattering, 1 H NMR Spectroscopy, and Hydrolysis Reaction of Carbonate as a Probe. Langmuir.

[68] Maitra A. (1984). Determination of size parameters of water-Aerosol OT-oil reverse micelles from their nuclear magnetic resonance data. J. Phys. Chem..

[69] Lépori C.M.O., Correa N.M., Silber J.J., Vaca Chávez F., Falcone R.D. (2019). Interfacial properties modulated by the water confinement in reverse micelles created by the ionic liquid-like surfactant bmim-AOT. Soft Matter.

[70] Lépori C.M.O., Correa N.M., Silber J.J., Falcone R.D., López-López M., Moyá M.L. (2020). Influence of the AOT Counterion Chemical Structure on the Generation of Organized Systems. Langmuir.

[71] Lépori C.M.O., Silber J.J., Falcone R.D., Correa N.M. (2017). Improvement of the amphiphilic properties of a dialkyl phosphate by creation of a protic ionic liquid-like surfactant. RSC Adv..

[72] Gupta S., Moulik S.P. (2008). Biocompatible Microemulsions and Their Prospective Uses in Drug Delivery. J. Pharm. Sci..

[73] Kogan A., Garti N. (2006). Microemulsions as transdermal drug delivery vehicles. Adv. Colloid Interface Sci..

[74] Fadel O., Girard L., Gomes Rodrigues D., Bauduin P., Le Goff X., Rossignol-Castera A., L’Hermitte A., Diat O. (2017). Micellization in vegetable oils: A structural characterisation. Colloids Surf. B Biointerfaces.

[75] Sun X., Bandara N. (2019). Applications of reverse micelles technique in food science: A comprehensive review. Trends Food Sci. Technol..

[76] Mitsou E., Xenakis A., Zoumpanioti M. (2017). Oxidation Catalysis by Enzymes in Microemulsions. Catalysts.

[77] Xenakis A., Papadimitriou V., Sotiroudis T.G. (2010). Colloidal structures in natural oils. Curr. Opin. Colloid Interface Sci..

[78] Majhi P.R., Moulik S.P. (1999). Physicochemical studies on biological macro- and microemulsions VI: Mixing behaviors of Eucalyptus oil, Water and polyoxyethylene sorbitan monolaurate (Tween 20) assisted by n-Butanol or Cinnamic Alcohol. J. Dispers. Sci. Technol..

[79] Mukhopadhyay L., Mitra N., Bhattacharya P.K., Moulik S.P. (1997). Thermodynamics of Formation of Biological Microemulsion (with Cinnamic Alcohol, Aerosol OT, Tween 20, and Water) and Kinetics of Alkaline Fading of Crystal Violet in Them. J. Colloid Interface Sci..

[80] Papadimitriou V., Sotiroudis T.G., Xenakis A. (2007). Olive Oil Microemulsions:â€‰ Enzymatic Activities and Structural Characteristics. Langmuir.

[81] Tzika E.D., Christoforou M., Pispas S., Zervou M., Papadimitriou V., Sotiroudis T.G., Leontidis E., Xenakis A. (2011). Influence of Nanoreactor Environment and Substrate Location on the Activity of Horseradish Peroxidase in Olive Oil Based Water-in-Oil Microemulsions. Langmuir.

[82] Papadimitriou V., Tzika E.D., Pispas S., Sotiroudis T.G., Xenakis A. (2011). Microemulsions based on virgin olive oil: A model biomimetic system for studying native oxidative enzymatic activities. Colloids Surf. A Physicochem. Eng. Asp..

[83] Papadimitriou V., Sotiroudis T.G., Xenakis A. (2005). Olive oil microemulsions as a biomimetic medium for enzymatic studies: Oxidation of oleuropein. J. Am. Oil Chem. Soc..

[84] Kyriazi A., Papadimitriou V., Sotiroudis T.G., Xenakis A. (2013). Development and characterization of a digestion model based on olive oil microemulsions. Eur. J. Lipid Sci. Technol..

[85] Chatzidaki M.D., Papadimitriou K., Alexandraki V., Tsirvouli E., Chakim Z., Ghazal A., Mortensen K., Yaghmur A., Salentinig S., Papadimitriou V. (2016). Microemulsions as Potential Carriers of Nisin: Effect of Composition on Structure and Efficacy. Langmuir.

[86] Mitsou E., Tavantzis G., Sotiroudis G., Ladikos D., Xenakis A., Papadimitriou V. (2016). Food grade water-in-oil microemulsions as replacement of oil phase to help process and stabilization of whipped cream. Colloids Surf. A Physicochem. Eng. Asp..

[87] Mitsou E., Dupin A., Sassi A.H., Monteil J., Sotiroudis G.T., Leal-Calderon F., Xenakis A. (2019). Hydroxytyrosol encapsulated in biocompatible water-in-oil microemulsions: How the structure affects in vitro absorption. Colloids Surf. B Biointerfaces.

[88] Mitsou E., Pletsa V., Sotiroudis G.T., Panine P., Zoumpanioti M., Xenakis A. (2020). Development of a microemulsion for encapsulation and delivery of gallic acid. The role of chitosan. Colloids Surf. B Biointerfaces.

[89] Papadimitriou V., Pispas S., Syriou S., Pournara A., Zoumpanioti M., Sotiroudis T.G., Xenakis A. (2008). Biocompatible Microemulsions Based on Limonene:  Formulation, Structure, and Applications. Langmuir.

[90] Chatzidaki M.D., Balkiza F., Gad E., Alexandraki V., Avramiotis S., Georgalaki M., Papadimitriou V., Tsakalidou E., Papadimitriou K., Xenakis A. (2019). Reverse micelles as nano-carriers of nisin against foodborne pathogens. Part II: The case of essential oils. Food Chem..

[91] Oyarzun M., Oliva G., Falcone R.D., Pavez P. (2020). Characterization of Anionic Reverse Micelles Formulated on Biobased Solvents as Replacing Conventional Nonpolar Organic Solvents. ACS Sustain. Chem. Eng..

[92] Girardi V.R., Silber J.J., Mariano Correa N., Darío Falcone R. (2014). The use of two non-toxic lipophilic oils to generate environmentally friendly anionic reverse micelles without cosurfactant. Comparison with the behavior found for traditional organic non-polar solvents. Colloids Surf. A Physicochem. Eng. Asp..

[93] Molina P.G., Silber J.J., Correa N.M., Sereno L. (2007). Electrochemistry in AOT reverse micelles. A powerful technique to characterize organized media. J. Phys. Chem. C.

[94] Sabaragamuwe S.G., Conti D., Puri S.R., Andreu I., Kim J. (2019). Single-Entity Electrochemistry of Nanoemulsion: The Nanostructural Effect on Its Electrochemical Behavior. Anal. Chem..

[95] Kalaitzaki A., Papanikolaou N.E., Karamaouna F., Dourtoglou V., Xenakis A., Papadimitriou V. (2015). Biocompatible Colloidal Dispersions as Potential Formulations of Natural Pyrethrins: A Structural and Efficacy Study. Langmuir.

[96] Rokosik E., Siger A., Rudzińska M., Dwiecki K. (2020). Antioxidant activity and synergism of canolol and α-tocopherol in rapeseed oil is affected by the presence of phospholipid association colloids. LWT.

[97] Rokosik E., Siger A., Rudzińska M., Siejak P., Dwiecki K. (2020). Formation of Phospholipid Association Colloids in Rapeseed Oil and Their Effect on Lipid Autoxidation in the Presence of Sinapic and Ferulic Acid. Eur. J. Lipid Sci. Technol..

[98] Garti N., Yaghmur A., Leser M.E., Clement V., Watzke H.J. (2001). Improved Oil Solubilization in Oil/Water Food Grade Microemulsions in the Presence of Polyols and Ethanol. J. Agric. Food Chem..

[99] Chatzidaki M.D., Papavasileiou K.D., Papadopoulos M.G., Xenakis A. (2017). Reverse Micelles As Antioxidant Carriers: An Experimental and Molecular Dynamics Study. Langmuir.

[100] Chatzidaki M.D., Mateos-Diaz E., Leal-Calderon F., Xenakis A., Carrière F. (2016). Water-in-oil microemulsions versus emulsions as carriers of hydroxytyrosol: An in vitro gastrointestinal lipolysis study using the pHstat technique. Food Funct..

[101] Chatzidaki M.D., Arik N., Monteil J., Papadimitriou V., Leal-Calderon F., Xenakis A. (2016). Microemulsion versus emulsion as effective carrier of hydroxytyrosol. Colloids Surf. B Biointerfaces.

[102] Bubic Pajic N., Nikolic I., Mitsou E., Papadimitriou V., Xenakis A., Randjelovic D., Dobricic V., Smitran A., Cekic N., Calija B. (2018). Biocompatible microemulsions for improved dermal delivery of sertaconazole nitrate: Phase behavior study and microstructure influence on drug biopharamaceutical properties. J. Mol. Liq..

[103] Nikolic I., Jasmin Lunter D., Randjelovic D., Zugic A., Tadic V., Markovic B., Cekic N., Zivkovic L., Topalovic D., Spremo-Potparevic B. (2018). Curcumin-loaded low-energy nanoemulsions as a prototype of multifunctional vehicles for different administration routes: Physicochemical and in vitro peculiarities important for dermal application. Int. J. Pharm..

[104] Nikolic I., Mitsou E., Damjanovic A., Papadimitriou V., Antic-Stankovic J., Stanojevic B., Xenakis A., Savic S. (2020). Curcumin-loaded low-energy nanoemulsions: Linking EPR spectroscopy-analysed microstructure and antioxidant potential with in vitro evaluated biological activity. J. Mol. Liq..

[105] Fubini B., Gasco M.R., Gallarate M. (1988). Microcalorimetric study of microemulsions as potential drug delivery systems. I. Evaluation of enthalpy in the absence of any drug. Int. J. Pharm..

[106] Attwood D., Mallon C., Taylor C.J. (1992). Phase studies on oil-in-water phospholipid microemulsions. Int. J. Pharm..

[107] Zhang X., Chen Y., Liu J., Zhao C., Zhang H. (2012). Investigation on the structure of water/AOT/IPM/alcohols reverse micelles by conductivity, dynamic light scattering, and small Angle X-ray Scattering. J. Phys. Chem. B.

[108] Kundu K., Paul B.K. (2013). Physicochemical investigation of biocompatible mixed surfactant reverse micelles: III. Aqueous NaCl solubilization, thermodynamic parameters of desolubilization process and conductometric studies. J. Surfactants Deterg..

[109] Bardhan S., Kundu K., Saha S.K., Paul B.K. (2013). Physicochemical investigation of mixed surfactant microemulsions: Water solubilization, thermodynamic properties, microstructure, and dynamics. J. Colloid Interface Sci..

[110] Liu J.X., Zhang X.G., Zhang H.J. (2014). Water/AOT/IPM/alcohol reverse microemulsions: Influence of salts and nonionic surfactants on structure and percolation behavior. J. Chem. Thermodyn..

[111] Girardi V.R., Silber J.J., Falcone R.D., Correa N.M. (2018). Micropolarity and Hydrogen-Bond Donor Ability of Environmentally Friendly Anionic Reverse Micelles Explored by UV/Vis Absorption of a Molecular Probe and FTIR Spectroscopy. ChemPhysChem.

[112] Das S., Mukherjee I., Paul B.K., Ghosh S. (2014). Physicochemical Behaviors of Cationic Gemini Surfactant (14-4-14) Based Microheterogeneous Assemblies. Langmuir.

[113] Kalaitzaki A., Poulopoulou M., Xenakis A., Papadimitriou V. (2014). Surfactant-rich biocompatible microemulsions as effective carriers of methylxanthine drugs. Colloids Surf. A Physicochem. Eng. Asp..

[114] Chatzidaki M.D., Mitsou E., Yaghmur A., Xenakis A., Papadimitriou V. (2015). Formulation and characterization of food-grade microemulsions as carriers of natural phenolic antioxidants. Colloids Surf. A Physicochem. Eng. Asp..

[115] Guo J., Zhang L., Wang Y., Liu T., Gu X. (2019). Microstructural transitions in β-carotene loaded nonionic microemulsions upon aqueous phase dilution. Colloids Surf. A Physicochem. Eng. Asp..

[116] Odella E., Falcone R.D., Ceolín M., Silber J.J., Correa N.M. (2018). Structural Characterization of Biocompatible Reverse Micelles Using Small-Angle X-ray Scattering, 31P Nuclear Magnetic Resonance, and Fluorescence Spectroscopy. J. Phys. Chem. B.

[117] Odella E., Falcone R.D., Silber J.J., Correa N.M. (2016). Nanoscale Control Over Interfacial Properties in Mixed Reverse Micelles Formulated by Using Sodium 1,4-bis-2-ethylhexylsulfosuccinate and Tri-n-octyl Phosphine Oxide Surfactants. ChemPhysChem.

[118] Mitsou E., Kalogianni E.P., Georgiou D., Stamatis H., Xenakis A., Zoumpanioti M. (2019). Formulation and Structural Study of a Biocompatible Water-in-Oil Microemulsion as an Appropriate Enzyme Carrier: The Model Case of Horseradish Peroxidase. Langmuir.

[119] Mithila B., Varsha P. (2020). Microemulsion assisted transdermal delivery of a hydrophilic anti-osteoporotic drug: Formulation, in vivo pharmacokinetic studies, in vitro cell osteogenic activity. J. Appl. Pharm. Sci..

[120] Radwan S.A.A., El-Maadawy W.H., ElMeshad A.N., Shoukri R.A., Yousry C. (2020). Impact of Reverse Micelle Loaded Lipid Nanocapsules on the Delivery of Gallic Acid into Activated Hepatic Stellate Cells: A Promising Therapeutic Approach for Hepatic Fibrosis. Pharm. Res..

[121] Ye R., Ni M., Chen H., Li S. (2020). Synthesis of mesoporous nickel–titanium-trimesic acid inorganic–organic hybrid composite in ionic liquid microemulsions for adsorption of rhodamine B from aqueous solution. Mater. Express.

[122] Ye R., Ni M., Chen H., Li S. (2020). Photochemical synthesis of silver nanoparticles in H 2 O/Triton X-100/[Bmim]PF 6 ionic liquid microemulsions and their antimicrobial activity. Mater. Express.

[123] Moniruzzaman M., Kamiya N., Nakashima K., Goto M. (2008). Water-in-ionic liquid microemulsions as a new medium for enzymatic reactions. Green Chem..

[124] Moniruzzaman M., Kamiya N., Goto M. (2009). Biocatalysis in Water-in-Ionic Liquid Microemulsions: A Case Study with Horseradish Peroxidase. Langmuir.

[125] Piekart J., Łuczak J. (2015). Transport properties of aqueous ionic liquid microemulsions: Influence of the anion type and presence of the cosurfactant. Soft Matter.

[126] Lian Y., Zhao K. (2011). Study of micelles and microemulsions formed in a hydrophobic ionic liquid by a dielectric spectroscopy method. I. Interaction and percolation. Soft Matter.

[127] Chen Z., Nozaki R. (2012). Dielectric spectroscopy study on ionic liquid microemulsion composed of water, TX-100, and BmimPF 6. J. Chem. Phys..

[128] Chen K., Zhao K. (2014). Dielectric analysis of the [Bmim][PF6]/TX-100/ethyleneglycol nonaqueous microemulsions: Microstructures and percolation. Colloids Surf. A Physicochem. Eng. Asp..

[129] Piekart J., Łuczak J. (2016). Transport properties of microemulsions with ionic liquid apolar domains as a function of ionic liquid content. RSC Adv..

[130] Seth D., Chakraborty A., Setua P., Sarkar N. (2006). Interaction of Ionic Liquid with Water in Ternary Microemulsions (Triton X-100/Water/1-Butyl-3-methylimidazolium Hexafluorophosphate) Probed by Solvent and Rotational Relaxation of Coumarin 153 and Coumarin 151. Langmuir.

[131] Bilgili H., Bürger M., Stubenrauch C., Porada J.H. (2016). About the nanostructure of the ternary system water—[BMIm]PF 6—TX-100. J. Colloid Interface Sci..

[132] Gao Y., Han S., Han B., Li G., Shen D., Li Z., Du J., Hou W., Zhang G. (2005). TX-100/Water/1-Butyl-3-methylimidazolium Hexafluorophosphate Microemulsions. Langmuir.

[133] Kaur M., Singh G., Kaur A., Sharma P.K., Kang T.S. (2019). Thermally Stable Ionic Liquid-Based Microemulsions for High-Temperature Stabilization of Lysozyme at Nanointerfaces. Langmuir.

[134] Seth D., Chakraborty A., Setua P., Sarkar N. (2007). Interaction of ionic liquid with water with variation of water content in 1-butyl-3-methyl-imidazolium hexafluorophosphate ([bmim] [P F6])/TX-100/water ternary microemulsions monitored by solvent and rotational relaxation of coumarin 153 and coumarin 490. J. Chem. Phys..

[135] Anjum N., Stubenrauch C., Mourchid A., Guedeau-Boudeville M.A., Stubenrauch C., Mourchid A. (2009). Phase behavior and microstructure of microemulsions containing the hydrophobic ionic liquid 1-butyl-3-methylimidazolium hexafluorophosphate. J. Phys. Chem. B.

[136] Shang W., Kang X., Ning H., Zhang J., Zhang X., Wu Z., Mo G., Xing X., Han B. (2013). Shape and Size Controlled Synthesis of MOF Nanocrystals with the Assistance of Ionic Liquid Mircoemulsions. Langmuir.

[137] Fu C., Zhou H., Peng W., Chen J., Kuang Y. (2008). Comparison of electrodeposition of silver in ionic liquid microemulsions. Electrochem. Commun..

[138] Gao Y., Li N., Zheng L., Zhao X., Zhang S., Han B., Hou W., Li G. (2006). A cyclic voltammetric technique for the detection of micro-regions of bmimPF6/Tween 20/H 2 O microemulsions and their performance characterization by UV-Visspectroscopy. Green Chem..

[139] Moniruzzaman M., Kamiya N., Nakashima K., Goto M. (2008). Formation of Reverse Micelles in a Room-Temperature Ionic Liquid. ChemPhysChem.

[140] Porada J.H., Mansueto M., Laschat S., Stubenrauch C. (2017). Microemulsions with hydrophobic ionic liquids: Influence of the structure of the anion. J. Mol. Liq..

[141] Zhou G.-P., Zhang Y., Huang X.-R., Shi C.-H., Liu W.-F., Li Y.-Z., Qu Y.-B., Gao P.-J. (2008). Catalytic activities of fungal oxidases in hydrophobic ionic liquid 1-butyl-3-methylimidazolium hexafluorophosphate-based microemulsion. Colloids Surf. B Biointerfaces.

[142] Cheng S., Fu X., Liu J., Zhang J., Zhang Z., Wei Y., Han B. (2007). Study of ethylene glycol/TX-100/ionic liquid microemulsions. Colloids Surf. A Physicochem. Eng. Asp..

[143] Xue L., Qiu H., Li Y., Lu L., Huang X., Qu Y. (2011). A novel water-in-ionic liquid microemulsion and its interfacial effect on the activity of laccase. Colloids Surf. B Biointerfaces.

[144] Safavi A., Maleki N., Farjami F. (2010). Phase behavior and characterization of ionic liquids based microemulsions. Colloids Surf. A Physicochem. Eng. Asp..

[145] Damarla K., Rachuri Y., Suresh E., Kumar A. (2018). Nanoemulsions with All Ionic Liquid Components as Recyclable Nanoreactors. Langmuir.

[146] Cheng S., Zhang J., Zhang Z., Han B. (2007). Novel microemulsions: Ionic liquid-in-ionic liquid. Chem. Commun..

[147] Rao V.G., Banerjee C., Ghosh S., Mandal S., Kuchlyan J., Sarkar N. (2013). A Step toward the Development of High-Temperature Stable Ionic Liquid-in-Oil Microemulsions Containing Double-Chain Anionic Surface Active Ionic Liquid. J. Phys. Chem. B.

[148] Banerjee C., Mandal S., Ghosh S., Kuchlyan J., Kundu N., Sarkar N. (2013). Unique Characteristics of Ionic Liquids Comprised of Long-Chain Cations and Anions: A New Physical Insight. J. Phys. Chem. B.

[149] Banerjee C., Kundu N., Ghosh S., Mandal S., Kuchlyan J., Sarkar N. (2013). Fluorescence Resonance Energy Transfer in Microemulsions Composed of Tripled-Chain Surface Active Ionic Liquids, RTILs, and Biological Solvent: An Excitation Wavelength Dependence Study. J. Phys. Chem. B.

[150] Wang C., Zhu J., Zhang D., Yang Y., Zheng L., Qu Y., Yang X., Cui X. (2018). Ionic liquid—Microemulsions assisting in the transdermal delivery of Dencichine: Preparation, in-vitro and in-vivo evaluations, and investigation of the permeation mechanism. Int. J. Pharm..

[151] Islam M.R., Chowdhury M.R., Wakabayashi R., Kamiya N., Moniruzzaman M., Goto M. (2020). Ionic liquid-in-oil microemulsions prepared with biocompatible choline carboxylic acids for improving the transdermal delivery of a sparingly soluble drug. Pharmaceutics.

[152] Dib N., Silber J.J., Correa N.M., Falcone R.D. (2019). Combination of a protic ionic liquid-like surfactant and biocompatible solvents to generate environmentally friendly anionic reverse micelles. New J. Chem..

[153] Dib N., Silber J.J., Correa N.M., Falcone R.D. (2020). Imim-DEHP reverse micelles investigated with two molecular probes reveals how are the interfacial properties and the coordination behavior of the surfactant. J. Mol. Liq..

[154] Dib N., Falcone R.D., Acuña A., García-Río L. (2020). The ionic liquid-surfactant bmim-AOT and nontoxic lipophilic solvents as components of reverse micelles alternative to the traditional systems. A study by 1H NMR spectroscopy. J. Mol. Liq..

[155] Dib N., Falcone R.D., García-Río L. (2020). Hydrolysis Reactions of Two Benzoyl Chlorides as a Probe to Investigate Reverse Micelles Formed by the Ionic Liquid-Surfactant bmim–AOT. J. Org. Chem..

